# Pareto-optimized stacked ensemble machine learning framework for predicting bearing capacity of driven piles from static load test data

**DOI:** 10.1038/s41598-026-43660-z

**Published:** 2026-04-02

**Authors:** Mohamed Abdellatief, Amr ElNemr, Ayman Altahrany

**Affiliations:** 1 Department of Civil Engineering, Higher Future Institute of Engineering and Technology in Mansoura, Mansoura, Egypt; 2 Civil Engineering Department, German University in Cairo (GUC), Cairo, 11835 Egypt; 3 Structural Engineering Department, Faculty of Engineering, Mansoura University, Mansoura, Egypt

**Keywords:** Pile bearing capacity, Geotechnical design, Machine learning, Stacked ensemble model, Pareto multi-objectives optimization, Engineering, Solid Earth sciences

## Abstract

Since traditional empirical methods frequently fail to capture complex soil–pile interactions, accurately evaluating the bearing capacity of driven piles remains a critical yet difficult task in geotechnical engineering. In this regard, this study proposes a novel machine learning framework for predicting the pile bearing capacity (PBC) of driven piles using a stacking guided by a multi-objective Pareto optimization approach. Using a comprehensive dataset that includes 472 records from the literature, the framework combines random forest (RF), K-nearest neighbor (KNN), and extreme gradient boosting (XGBoost) to increase predictive accuracy and generalizability. The dataset consists of a range of geological, geometric, and loading conditions, in addition to explanatory input features from the standard penetration test (SPT) dataset, such as pile diameter, soil depth, different soil layers, and the SPT-N values. Model interpretability is achieved through Shapley Additive exPlanations (SHAP) and Partial Dependence Plots (PDP), revealing key influencers such as pile diameter and SPT-N values along the shaft and tip. The stacking model improved predictive accuracy over the respective base-level models, using a Pareto-optimized stacking model, achieving a coefficient of determination of 0.9471. It also had reduced mean squared error (MSE) and mean absolute error (MAE) on the testing dataset as compared to the base-level models. Sensitivity and parametric analyses of the PBC confirmed that the model calculations were significant and demonstrated that pile diameter and soil layer thicknesses influence PBC. Finally, this approach provides geotechnical engineers with an accurate and interpretable tool to predict PBC, supporting safer and more cost-effective pile foundation designs in complex soil conditions.

**Supplementary Information:**

The online version contains supplementary material available at 10.1038/s41598-026-43660-z.

## Introduction

Pile foundations are used to transfer building loads to deeper and generally more suitable soil and/or rock layers^1,2^. Because of their stability, pile systems have become one of the most commonly used foundation systems in the construction industry. One of the key parameters for the design of a pile foundation is pile bearing capacity (PBC), because knowing the PBC of piles is imperative^1,3^. Pile foundations can support only vertical loads; however, properly installed piles are well capable of supporting loading to some degree from horizontal forces, especially those forces that can result from shifting weak surface soils^4,5^. Furthermore, foundation work is often the costliest portion of a construction project^6^; hence, determining the proper type of foundation along with a reliable method of determining the PBC is critical to keeping overall construction costs low^7,8^. Accurate PBC predictions support overall cost efficiency and a structurally safe building. On the other hand, inaccurate predictions could lead to severe structural failure. Several methods have been employed to evaluate the PBC, such as analytical methods^9,10^, experimental investigations^11–13^, empirical formulas^14,15^, numerical modeling simulations^14,16^, and hybrid methods that combine numerical modeling and physical models^17,18^. These approaches have made significant strides in advancing the understanding of load transfer mechanisms through different pile types, particularly composite piles. On the other hand, the complex nature of soil–pile interactions and the variability of subsurface conditions are natural limitations of these approaches due to a lack of standardization and reliable accuracy. From the various techniques, the static load test (SLT) is likely the most meaningful and direct method for assessing the PBC. The SLT involves applying vertical loads to a test pile in increments and observing the settlement of that pile over time to provide performance data taken in the field for use in improving a more empirical or analytical model^17,19,20^. The SLT provides a high degree of accuracy, but it can also be limited by cost, duration, and logistical constraints (e.g., remote sites, large diameter piles, etc.).

While traditional approaches are taking several developments, machine learning (ML) techniques have gained more attention due to their high levels of precision and accuracy in PBC prediction^7,8,13,15,21^. These ML techniques have enabled effective analysis of complex geotechnical datasets and improved pile behavior modeling by aggregating varying soil and loading conditions^3–5^. Table 1 summarizes previous studies according to pile type, training dataset, and modeling technique. The broad studies in the data that were collected mostly used artificial neural networks (ANN) to resolve the non-linear relationships underlying PBC prediction. Chan et al.^22^ applied ANN to predict driven pile load capacities based on data from 68 pile load tests. Although their study was constrained by a comparatively small dataset, Lee and Lee^23^ used an ANN to model load displacement responses from pile tests. Teh et al.^24^ also used ANN to predict the PBC, but their results were limited by the dataset.

**Table 1:** Application of ML techniques in PBC prediction.

Models	Datasets	Test type	Pile type	Refs.
ANN	68	DLT	Driven piles	^22^
ANN	38	Pile load test	Undrained lateral load capacity of piles	^30^
ANN	37	DLT	Precast reinforced concrete piles	^24^
GRNN	59	Pile load test	Driven piles	^31^
GPR	116	Pile load test	Precast driven piles	^32^
ANN	104	DLT	Driven pipe piles	^33^
Genetic algorithm-based ANN	50	DLT	Precast concrete piles	^26^
GPR	296	DLT	Precast driven piles	^34^
MARS, RBFNN	2314	SLT	Precast reinforced concrete pile	^28^
KNR, XGBoost, and (DNN)	214	DLT	Prebored and precast piles	^7^
DNN, CNN, RNN, LSTM	257	DLT	Driven piles	^29^
KRR	150	Cases from numerical analysis	Modeled pile	^35^
XGBoost with Bayesian optimization	138	cases from the literature	Rock-socketed piles	^36^
Decision tree, random forest, gradient boosted tree	80	Cone penetration test (CPT)	Ultimate capacity of piles	^37^
Pareto optimization	1178	SPT-N values and SLT	Precast pre-stressed centrifugal concrete (PHC) piles	^8^
Pareto-optimized stacked ML (RF, KNN, and XGBoost)	472	SLT	Driven piles	This study

While the ANN is limited by the dataset, researchers^25–29^ began to evaluate various ML methods. Abu-Kiefa^25^ developed a general regression neural network (GRNN), which predicts the driven pile on cohesive soil. Further progress was made by integrating ML with metaheuristic optimization algorithms. For instance, Pal and Deswal^26^ implemented Gaussian process regression (GPR) with a dataset of 116 cases; their methodology demonstrated reasonable results, although these results were data-size reliant. Momeni et al.^26^ utilized genetic algorithms (GA) to improve the adaptability of ANN models, with respect to improving the model performance relative to the computational resource use. Further, Armaghani et al.^27^ optimized predictive accuracy by coupling particle swarm optimization (PSO) with ANN for rock-socketed piles, but success was achieved only when both models were able to find a common set of parameters. As a collective, these studies illustrate the evolution of ML-based methods using pile behaviour concepts, their potential, and limitations. For example, Pham et al.^28^ presented a complete evaluation of reinforced concrete piles using random forest (RF) and ANN with SLT datasets. They indicated an enhanced predictive performance with the use of ensemble methods. Kumar et al.^29^ tested several deep learning methods deep neural networks (DNN), convolutional neural networks (CNN), and long short-term memory (LSTM) networks, to predict driven pile bending behaviour. These studies demonstrate an increasing change towards hybrid and optimization-based predictive models to replicate the complexity of pile-soil interaction across diverse ranges of behavior (Table 1).

In recent years, ML has emerged as a promising alternative, offering enhanced capability to model complex geotechnical behaviors. However, the application of advanced ensemble learning, particularly stacked generalization (stacking), remains underexplored in the context of PBC prediction. Stacking, as a meta-learning approach, integrates the predictive strengths of multiple base learners—such as random forest (RF), K-nearest neighbor regression (KNN), and extreme gradient boosting (XGBoost)—to construct a more generalized and robust predictive model. Although this technique has been successfully applied in various geotechnical applications, including soil shear strength estimation^38^, slope stability analysis^39^, and geological interface detection^40^, its adoption in pile foundation design has been limited. Hence, this research aims to bridge that gap by implementing a stacking framework specifically designed for driven piles, augmented by multi-objective Pareto optimization to simultaneously enhance model accuracy, reduce error, and maintain model generalizability. A curated dataset comprising 472 SLT records was assembled, supporting the developed ML framework. These records encompass a diverse set of driven pile installations under varying geological, geometric, and loading conditions. Model interpretability, a crucial consideration in engineering applications, is achieved using Shapley Additive exPlanations (SHAP) and Partial Dependence Plots (PDP), providing transparency into the influential input features on the predicted capacity.

## Materials and methodologies

### Dataset preparation

In this study, subsurface soil conditions in Ha Nam province, Vietnam (Fig. S1), were assessed primarily using the SPT, as per the research project performed by Pham et al.^41^. SPT was selected, instead of the cone penetration test (CPT), due to the advantages of logistics, including cost and suitability for diverse soil conditions, in addition to the existence of considerable legacy datasets in regional engineering practice. Although SPT provides valuable soil resistance data, the data in very soft clays or in gravel layers were identified, and the authors mitigated data integrity concerns by validation and preprocessing steps, allowing reliable data as input for the ML framework. SPT procedures were conducted according to the Vietnamese Standard, *TCVN 9351:2012,* with respect to ASTM D1586-99. Thus, the full data collection conformed to standard procedures of data collection. Likewise, static pile load testing, which is necessary to establish PBC, also followed the *TCVN 9393:2012* protocol, and it can be compared to ASTM D1143-81. Loading for the load tests followed a slow, maintained load protocol that is suited for characterizing failure and settlement behavior in layered soils. There was a minimum seven-day resting period between installation and full testing, to minimize the pore water pressure dissipation and installation disturbance as suggested by Huynh et al.^42^, who suggested that resting periods are critical for reliable assessment of short-term PBC. Therefore, the study focused on short-term assessment of pile foundation performance (*one to four weeks after installation*) and neglected time-dependent changes to soil-pile interaction^42–44^. Soil properties obtained from SPT blow count data (N) recorded along each pile’s embedded length were used to predict PBC. To account for layered soil variability in^41^, additional sampling was conducted to ensure representative SPT data. Two key SPT indicators were used: (i) the average blow count along the pile shaft, and (ii) the average blow count at the pile tip, calculated over a zone extending 8D above and 3D below the pile tip (*where D is the pile diameter*). This averaging follows Meyerhof’s recommendation as expressed in Eq. (1)^45^ for deriving a representative N-value near the tip, as also utilized in the source dataset^41^. The depth of the water table was excluded as an input variable, since its effect is already accounted for in SPT blow counts. The PBC can be conceptually expressed as the sum of shaft resistance and end-bearing resistance using the general framework adapted for deep foundations^45^, as shown in Eq. (1):

1 $${\mathrm{PBC}} = Q_{u} = Q_{s} + Q_{p} = A_{s} \left( {c_{a} + K_{s} \sigma _{v}^{'} \tan \delta } \right) + A_{b} \left( {cN_{c} + \sigma _{v}^{'} N_{q} + 0.5\gamma ^{\prime}BN_{\gamma } } \right)$$

where $A_{s}$ is the shaft surface area; $c_{a}$ is the soil adhesion per unit area; $K_{s}$ is the earth pressure coefficient; $\sigma_{v}{\prime}$ is the average effective vertical stress along the shaft; $\delta$ is the friction angle between pile and soil; $A_{b}$ is the base cross-sectional area; $c$ is the soil cohesion at the tip; $\sigma_{v}{\prime}$ is the effective vertical stress at the pile tip; $\gamma^{\prime}$ is the effective unit weight of the soil; $B$ is the pile diameter, while $N_{c}$, $N_{q}$, and $N_{\gamma }$ are the bearing capacity factors dependent on $\phi$ and embedment effects. In practice, the shaft and tip components are frequently estimated using empirical SPT correlations, as performed in the original study^41^.

**Fig. 1:**
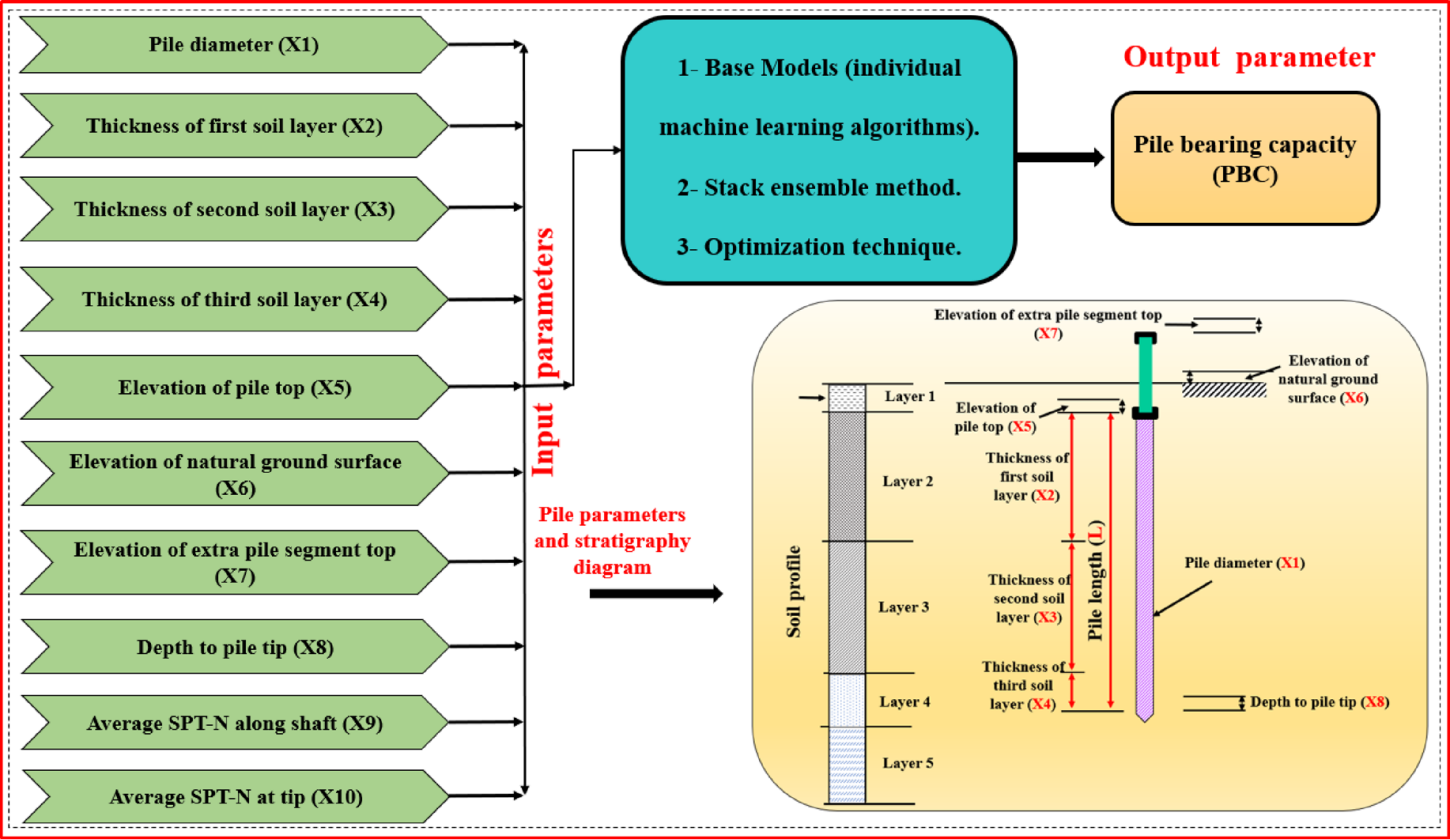
Schematic representation of the ML framework for predicting PBC, including the input parameters and soil stratigraphy. The output parameter (PBC) represents the ultimate axial bearing capacity obtained from SLT.

The experimental database used in this study was derived from pile load test results conducted on 472 individual SLT records at multiple test sites in Ha Nam province, Vietnam (see Supplementary Fig. S1 for the approximate location). The broad range of soil profiles included in the dataset adds confidence to the validity and generalisability of the proposed ML models compared to a smaller dataset used in previous studies^24,36^. The field testing was done in Ha Nam province, Vietnam, on pre-cast and closed-tip square-section reinforced concrete piles installed using a hydraulic pile press. Each static load test was carried out no sooner than 7 days after installation, and the axial load, which was either 100% or 200% of the design load, was applied in stages over a 6 to 24-h duration. PBC was evaluated based on two recognized criteria: (i) failure load was defined as the load where the settlement at a given load was at least five times settlement of the previous load level, and (ii) for nearly linear load–settlement relationships, the failure load was estimated at a point where the pile top settlement exceeded 10% of the pile diameter^46,47^. As indicated in Fig. 1, a comprehensive framework for predicting PBC using a Pareto-optimized stacking ensemble model is presented, which aligns well with the detailed dataset and methodology described. The ML model input variables from the SPT and SLT records comprise: pile diameter (X₁); thicknesses of soil layers (X₂–X₄); ground elevation and layer elevation (X₅–X₇); depth to pile tip (X₈); and average SPT-N values for given depth intervals (X₉, X₁₀). These input variables are relevant as existing literature demonstrates strong correlations between SPT-derived soil properties and predictions of PBC^45,48^. To leverage the potential of this dataset, a Pareto-optimized stacking ensemble learning framework is used to balance accuracy and generalization. Ensemble methods have been proven to provide better predictive performance in geotechnical applications^8,39,40,49^ when compared to traditional single-model approaches, particularly when working with multi-faceted and extensive datasets. By providing SPT-derived attributes into our model, a solution that ties tangible empirical testing to modern ML approaches is offered, enabling investigators to effectively improve pile design and, thus, minimize construction cost and improve safety on deep foundation projects. The implications of this will be beneficial for future site assessments, which often involve complex soil conditions, as is often faced throughout the region of Southeast Asia.

Table 2 provides the statistical summary of the input and output parameters for 472 SLT records, which provide necessary information on the variability and distribution, factors that will be important for creating an effective PBC prediction model. The descriptors included: pile diameter (X₁), thickness of soil layers (X₂–X₄), elevations (X₅–X₇), depth to pile tip (X₈), average SPT-N values along the shaft (X₉) and at the tip (X₁₀), and axial bearing capacity (Y). For instance, pile diameter (X₁) has an average of 0.36 m (Std = 0.05 m) with low variation (skewness = 1.72) shown by the data, representing a general, approximately the same design approach that dominated the study area. In contrast, the soil layers’ thicknesses showed more variation, particularly the first layer (X₂, average = 3.84 m); consistent with the different stratigraphy encountered in the Southeast Asian deposits^41,50^.

**Table 2:** The descriptive statistics of input features and output parameter (PBC).

Variables	Unit	Notation	Min	Mean	Std	Skewness	Max
Pile diameter	m	X1	0.3	0.36	0.05	1.72	0.4
Thickness of the first soil layer	m	X2	3.4	3.84	0.45	0.92	5.72
Thickness of the second soil layer	m	X3	1.5	6.82	1.55	− 2.21	8
Thickness of the third soil layer	m	X4	0	0.47	0.46	0.87	1.69
Elevation of pile top	m	X5	1.95	2.92	0.53	− 0.43	3.4
Elevation of the natural ground surface	m	X6	3.04	3.48	0.07	0.53	3.72
Elevation of the extra pile segment top	m	X7	1.03	2.98	0.56	− 0.44	4.05
Depth to pile tip	m	X8	8.3	12.98	1.83	− 1.25	16.09
Average SPT-N along the shaft	N-value	X9	5.6	10.12	2.33	− 0.69	15.41
Average SPT-N at tip	N-value	X10	4.38	6.94	0.53	− 1.23	7.75
Axial bearing capacity	kN	X11	407.2	947.96	373.74	0.06	1551

**Fig. 2:**
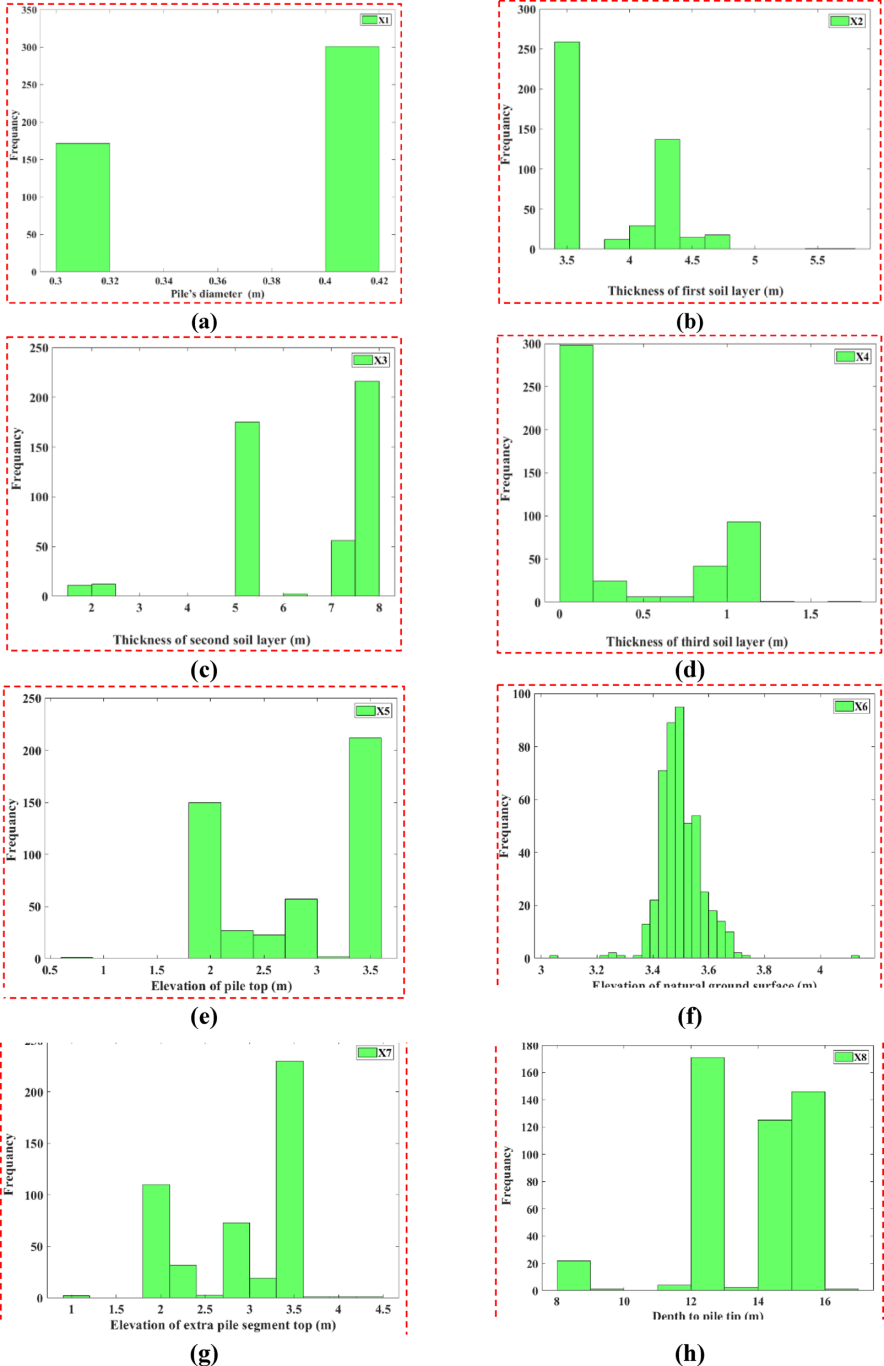
Histogram plots of the eight input parameters related to pile geometry and soil properties.

The histograms in Fig. 2 show the distributions of the eight geometric and stratigraphic input variables. The pile diameter (Fig. 2a) had a narrow spread, which indicates the design of the piles was fairly standard in Ha Nam province. The thicknesses of the first (Fig. 2b) and second (Fig. 2c) soil layers are right-skewed (*heavier left side*), which is consistent with the stratigraphic differences in soil properties and impact load transfer through the layers.

**Fig. 3:**
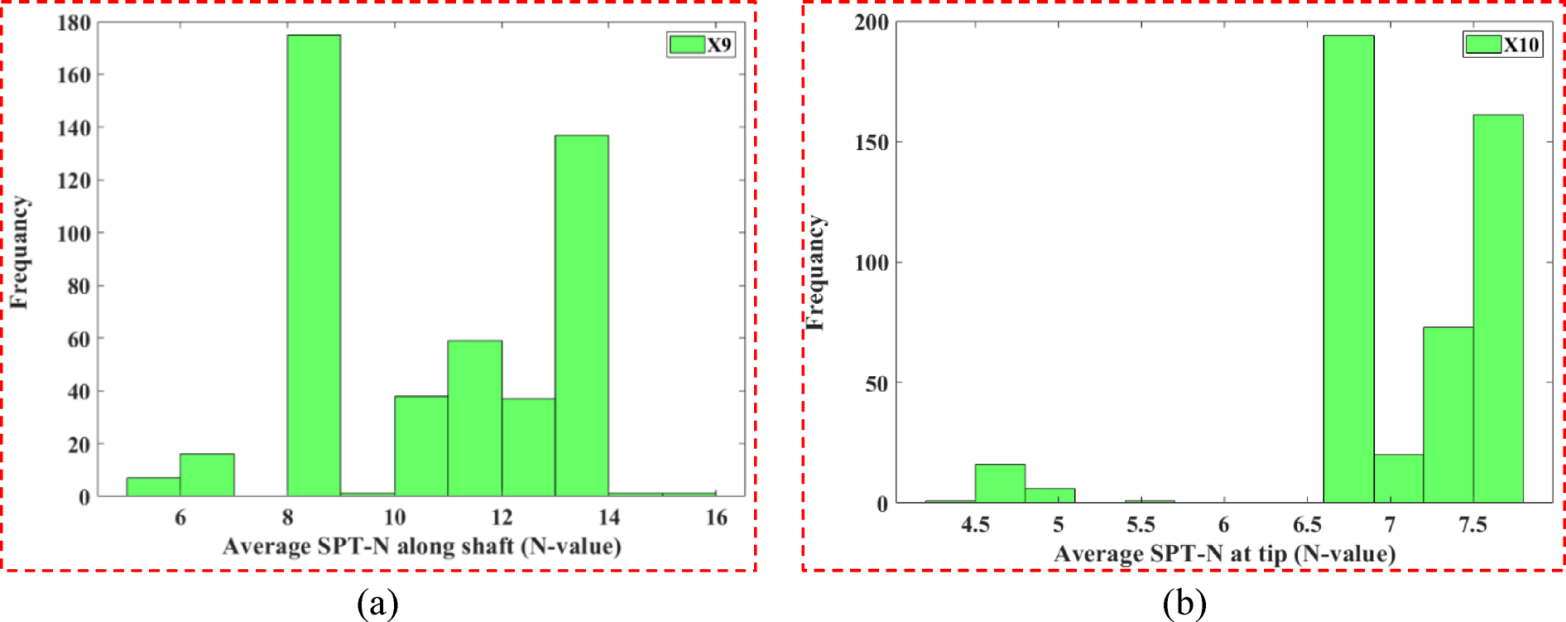
Frequency distribution histograms of two input variables: (**a**) average SPT-N along shaft (X9); (**b**) average SPT-N at tip (X10).

**Fig. 4:**
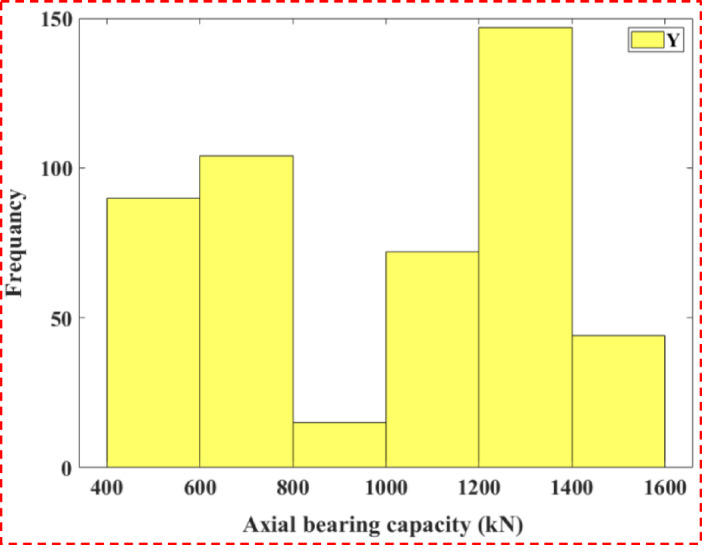
Frequency distribution histograms of the target (Y).

The thicknesses of the first (Fig. 2b) and second (Fig. 2c) soil layers are right-skewed (*heavier left side*), which is consistent with the stratigraphic differences in soil properties and impact load transfer through the layers. The third layer thickness (Fig. 2d) was more normally distributed, suggesting less predictability of the existence of multiple layers below the ground surface. Both the elevations of the pile top (Fig. 2e) and the natural ground surface (Fig. 2f) have tendencies towards central values through installation methods, whereas dealing with the depth to pile tip (Fig. 2g) and extra pile segment top elevation (Fig. 2h) were right-skewed; likely due to engineering difficulties. Previous studies have shown^35,36^ that some of the diversity in these parameters enhances the ML performance of capturing non-linear soil-pile interaction trends. Figure 3 provides histograms for both SPT-based variables, average N-value along the shaft (X9) and at the tip (X10). Though both variables are right-skewed, X9 peaks within 10–12 and X10 peaks within 6–6.5. The majority of piles are most likely to have resistance in these ranges, while the presence of higher N-values noted in the tails of the distributions undoubtedly relates to local dense strata that impart higher axial capacities. Figure 4 depicts the distribution for the output variable (Y), axial bearing capacity. The distribution is right-skewed with a peak between 1200–1400 kN, while the variation extends from 400 to 1600 kN, reflecting the cumulative effects of the geometric parameters and soil resistance on pile performance.

**Fig. 5:**
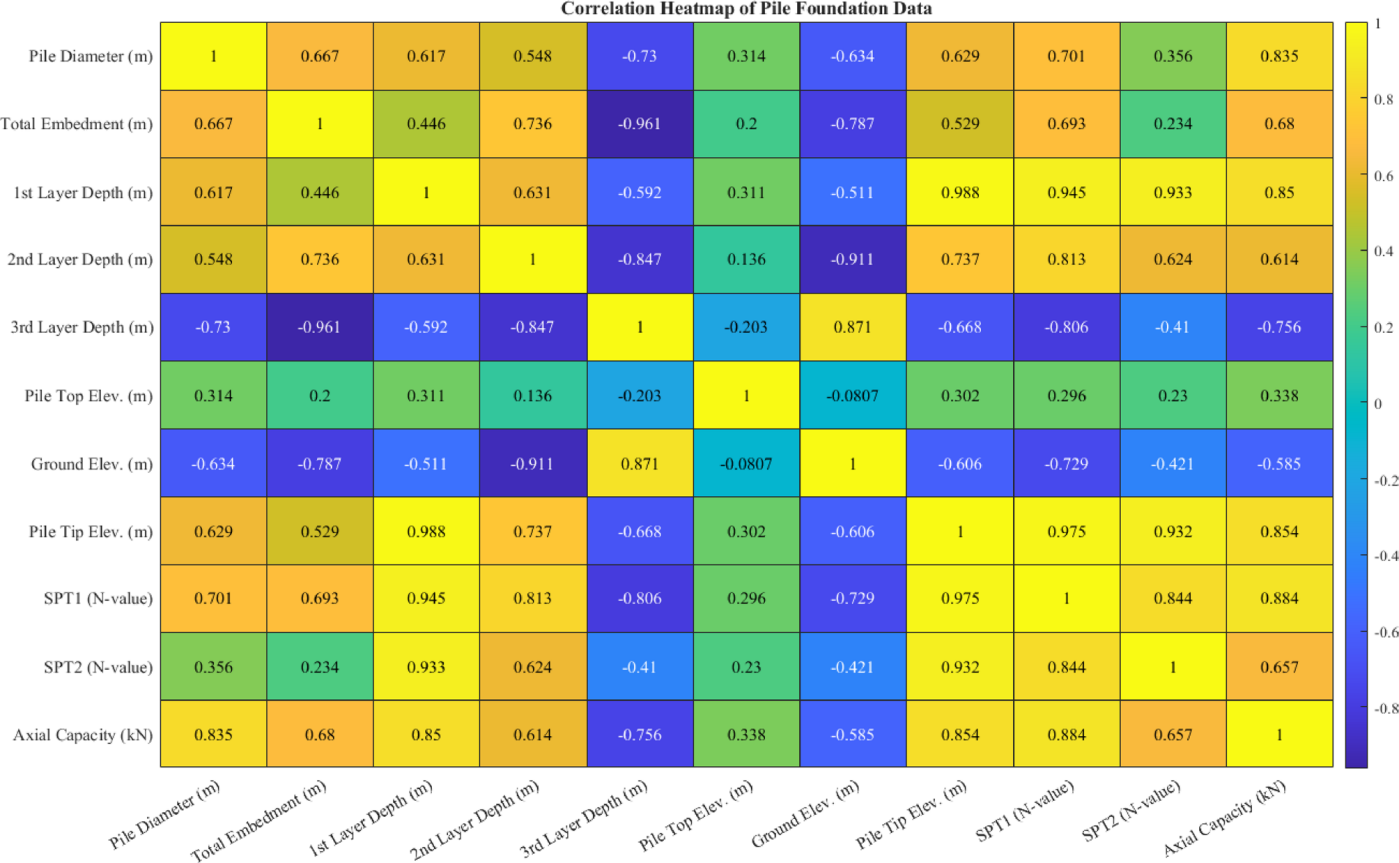
Correlation coefficient between features.

The heatmap of correlations shown in Fig. 5 provides the various correlations between input and output variables. Some notable cases include a strong positive correlation (r = 0.667) between pile diameter (X1) and total embedment (X2), indicating that larger diameter piles are generally installed to greater depths, particularly for more significant applications. Stronger positive correlations between SPT-N values (X9: 0.844, X10: 0.857) and the PBC (Y) indicate that the piles are predominantly governed by shaft and tip resistance. There were additional strong negative correlations with capacity for two of the elevation variables (X6: − 0.807, X7: − 0.858), which suggested lower elevation sites would have a tendency to yield higher capacities, potentially because more massive soils are found at depth. Further indication that some of the third-layer thickness (X8) may also account for variability in PBC is a moderate negative correlation (− 0.756). This indicates that thicker or deeper layers may represent weaker material that degrades capacity. Regardless, it is observed that both geometric factors (e.g., diameter, embedment depth) and geotechnical parameters (SPT-N values, stratigraphy) were important to accurately predict PBC. The observed variability and correlation patterns aligned with the most recent studies^8,17,42^ strengthen the justification for utilizing the proposed Pareto-optimized stacking ensemble framework to capture the non-linear, multivariate nature of soil–pile interactions.

### Methodology

#### Configuration of base models

The performance of 5 different ML models is explored for forecasting the bearing capacity of driven piles. The following ML models were used, which include different well-known algorithms: RF^21,51^, KNN^8^, XGBoost^51^, linear regression (LR)^52^, and support vector regression (SVR)^52,53^. Each model provided its own advantages to address the complexities of the problem. To ensure an unbiased selection process and best optimize the overall performance of the stacked ensemble model, each base model was tuned to their respective parameters, as discussed below.

**Fig. 6:**
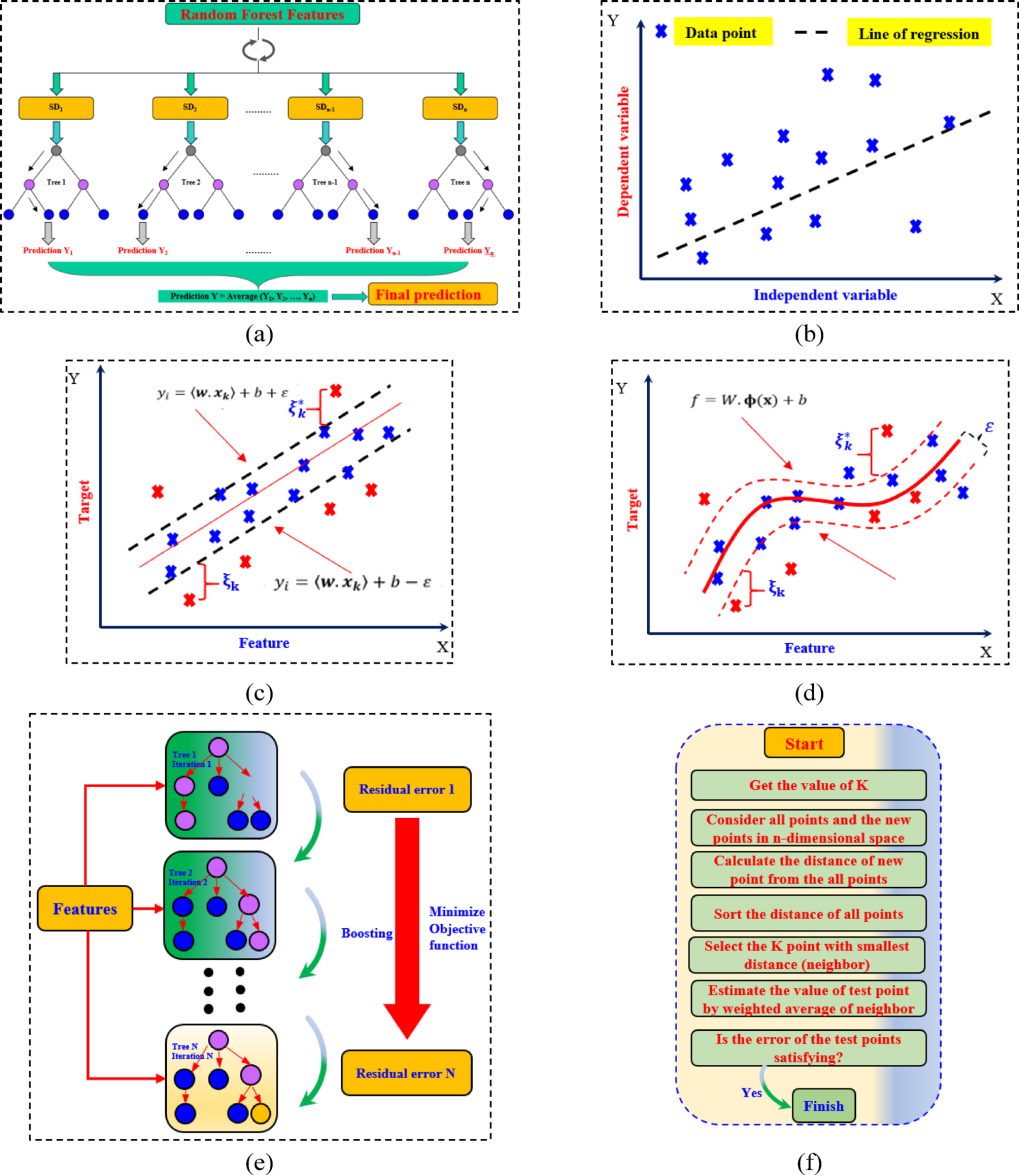
Schematic overview of the machine learning models for regression tasks. (**a**) RF, (**b**) LR, (**c**) Linear SVR, (**d**) Non-linear SVR, (**e**) XGBoost, and (**f**) KNN regression.

1. *Random forest (RF)* is a widely respected advanced method of regression landmarks that was seminally developed by Breiman^54^. The RF is developed by aggregating several independent decision trees, which have been drawn from a random process. The RF has many outstanding characteristics, including fast and flexible means of linking input and output variables. There are three steps of the RF method. First, construct the regression trees using your training set data. Second, take the average output from all the regression trees. Third, verify (check) the predicted outcomes using validation data. The training data is a second dataset that uses bootstrap samples derived from the initial training data. In this step, the data is dropped, and the data is replaced in the data set. The data that was dropped had various data points from the collected data- that is, the out-of-bag, or OOB data. The regression model is now trained using two-thirds of the points, and the validation of the model was completed by using the OOB data (as shown in Fig. 6a). The RF then repeats until your accuracy is satisfactory. While the RF uses OOB data to validate the habitual modelling process, it does so after the OOB was dropped from the original training data. In the end, the total error was calculated for all decision trees, resulting in an understanding of the exactness, and precision of each tree (Fig. 6a). It is essential to note from the RF regression method, it is only a helpful method because it minimized overfitting, maximized the prediction accuracy, and combined predictions from multiple decision trees which could express complex relationships.
2. *Linear regression (LR):* The LR is a supervised ML algorithm used to predict a dependent variable from a set of independent variables. Its goal is to create a linear relationship between the independent variables and the target variable^52^. This feature is reflected in its name as LR evaluates the relationship between independent and dependent variables to make predictions (Fig. 6b). The main advantage of predicting PBC with the LR technique is its simplicity and ease of use, which makes it straightforward to evaluate and easy to understand. Further information for the math model and equations for LR can be found in^55^.
3. *Support vector regression (SVR):* Several studies describe the SVR method thoroughly^51^. Therefore, a brief description of the SVR algorithm is proposed below. Assume a regression problem with the training data set (x_1_, y_1_), (x_2_, y_2_), (x_3_, y_3_) … (x_m_, y_m_) where m is the number of training patterns and x and y are the input and output values, respectively. The form of the generalized linear SVR regression function f(x):
  2 $${\text{f }}\left( {\mathrm{x}} \right){ } = { }\left( {{\text{w }} \times {\text{ x}}} \right){ } + {\text{ b }}$$
  where x is the input vector, w is the weight vector in the characteristic region, and b is the bias.

The SVR algorithm offers a new loss function L_ε_(y) which is called the ε-insensitive loss function. The ε-insensitive loss function allows SVR regression to utilize the margin property as its core concept. The ε-insensitive loss function states that if the error is less than ε, there is no prediction error in the model by definition.

3 $$L_{\varepsilon } \left( {y_{i} } \right) = \left\{ {\begin{array}{*{20}c} {\varepsilon ,} & {if\left| {y_{i} - f\left( {x_{j} ,w} \right)} \right| \le \varepsilon } \\ {\left| {y_{i} - f\left( {x_{j} ,w} \right)} \right| - \varepsilon ,} & {otherwise} \\ \end{array} } \right.$$

The goal of SVR is to find the flattest possible function, minimizing:

4 $$Minimize \frac{1}{2}\left\| {w^{2} } \right\|\quad Subjected\, to \left\{ {\begin{array}{*{20}c} {y_{k} - {\boldsymbol{w}} \cdot {\boldsymbol{x}}_{{\boldsymbol{k}}} - b \le \varepsilon } \\ {{\boldsymbol{w}} \cdot {\boldsymbol{x}}_{{\boldsymbol{k}}} + b - y_{k} \le \varepsilon } \\ \end{array} } \right.$$

To handle prediction errors outside the ε-margin, slack variables (ξ_k_, ξ_k_^*^) are introduced, resulting in the following objective:

5 $$Minimize \frac{1}{2}\left\| {w^{2} } \right\| + C\sum\nolimits_{k = 1}^{m} {\left( {\xi_{k} + \xi_{k}^{*} } \right)} \quad Subjected\, to \left\{ {\begin{array}{*{20}c} {y_{k} - {\boldsymbol{w}} \cdot {\boldsymbol{x}}_{{\boldsymbol{k}}} - b \le \varepsilon + \xi_{k} } \\ {{\boldsymbol{w}} \cdot {\boldsymbol{x}}_{{\boldsymbol{k}}} + b - y_{k} \le \varepsilon + \xi_{k}^{*} } \\ {\xi_{k} ,\xi_{k}^{*} \ge 0 } \\ \end{array} } \right.$$

where C > 0 is a regularization parameter that balances model complexity and tolerance to large errors. The problem can be solved in its dual form, involving Lagrange multipliers ($\alpha_{k}$,$\alpha_{k}^{*}$). Only data points with non-zero multipliers (called support vectors) contribute to the final prediction function:

6 $$f\left( x \right) = \sum\nolimits_{nsv} {\left( {\alpha_{k}^{*} - \alpha_{k} } \right)} + \sum\nolimits_{k = 1}^{m} {y_{k} \left( {x_{k}^{*} \cdot x} \right) + b}$$

where K($x_{k}^{*}$, $x$) is a kernel function, enabling SVR to handle nonlinear relationships by mapping input features into a higher-dimensional space. Popular kernel functions used in SVR include the linear kernel, polynomial kernel, and Gaussian (*radial basis function, RBF*) kernel. A significant advantage of SVR is that its optimization is a convex problem, ensuring a global optimum is always found. This makes it highly robust for both linear and nonlinear regression tasks. Figure 6c, d present the schematic diagram of linear-non-linear SVR along with ε-insensitive loss function.

4. *Extreme gradient boosting (XGBoost):* XGBoost is a state-of-the-art ensemble learning technique operating under the boosting methodology. Boosting is an iterative process where a series of weak learners, generally in the form of decision trees, are trained one after the other while attempting to obtain weight updates that model the previous weak learner (Fig. 6e). After the model tunes each iteration, the sample weights are updated assigning larger weights to samples which have been misclassified or had poor predicted values. The ultimate prediction model is formed by the aggregation of all the weak learners delivered from the XGBoost model, in which the outcome is a powerful ensemble model.

XGBoost is effective for regression tasks as it can model highly complex nonlinear relationships and enhance prediction accuracy through iterative refinement. XGBoost is considered the strongest form of ensemble learning for predicting PBC of driven piles due to its precise results, robustness, and manipulation of feature interactions. The gradient boosting (GB) framework was introduced by Friedman^56^, which iteratively reduces the residuals by applying new learners on the negative gradients from the loss function. The learning was described by,

7 $$G_{0} = \mathop {{\mathrm{argmin}}}\limits_{\gamma } \sum\nolimits_{i = 1}^{n} {L\left( {y_{i} ,\gamma } \right)}$$

8 $$G_{m} = G_{m - 1} + \mathop {{\mathrm{argmin}}}\limits_{{h_{m} \in H}} \left[ {L\left( {y_{i} ,G_{m - 1} \left( {x_{i} } \right) + h_{m} \left( {x_{i} } \right)} \right)} \right]$$

9 $$\nabla Loss = - \frac{{\partial L\left( {y_{i} ,G_{m - 1} \left( {x_{i} } \right)} \right)}}{{\partial G_{m - 1} \left( {x_{i} } \right)}}$$

where x_i_ is the ith variable, h_m_ is the base-function, G_0_ is the first weak-learner, L is the model’s loss function, y_i_ is the true outcome of the ith sample, γ is the optimal step length, G_m_ is the strong-learner, and ∇Loss is the negative gradient of the loss function. XGBoost was the first case of a GB presented by Chen and Guestrin^57^, which can be interpreted as an improvement upon GB models. Similar to GB, XGBoost improves upon the prediction performance of a weak learner iteratively, while optimizing the loss function by both first-order and second-order derivatives. A diagrammatic view of the XGBoost model is visually depicted in Fig. 6e. In addition, the loss function has regularization terms added to manage the complexity of the model to prevent overfitting. The goal function of the XGBoost algorithm can be articulated below:

10 $$F_{obj}^{\left( t \right)} = \mathop \sum \limits_{i = 1}^{n} L\left( {y_{i} ,\hat{y}_{i}^{{\left( {t - 1} \right)}} + f_{t} \left( {x_{i} } \right)} \right) + \Omega \left( {f_{t} } \right) + K$$

where F_obj_ is the objective function, L is the loss function, Ω is the regularization terms (L_1_ and L_2_), and K is the constant term.

5. *K-Nearest Neighbor (kNN):* The kNN is a simple and effective machine learning algorithm used for both classification and regression tasks^7,50^. The kNN identifies the k closest data points (neighbors) to a test sample, as calculated by distance metrics derived from the training data (the distances between the test sample and each training sample). These neighbors are then used to predict the output of the test sample^7^. During the training phase, the algorithm retains all training instances. When making a prediction using kNN, the algorithm calculates the distance between the test sample and each training sample (Fig. 6f). Typically, Euclidean, Manhattan, and Minkowski distances are used. In the present study, the Euclidean distance was calculated as follows:
  11 $$d = \sqrt {\sum\nolimits_{n = 1}^{h} {\left( {x_{testing,n} - x_{training,n} } \right)^{2} } }$$
  where: d is the distance between the test and training samples, and h is the number of dimensions (features) in the data.

The kNN algorithm proceeds in four main steps:

- Compute the Euclidean distance between the test sample and each sample in the training set.
- Sort the distances in ascending order and select the k nearest neighbors.
- Determine the most frequent class (for classification) or the average output (for regression) among the selected neighbors.
- Calculate the prediction accuracy by comparing the predicted values with actual values^7,58^.

#### Stacking-based ensemble framework

**Fig. 7:**
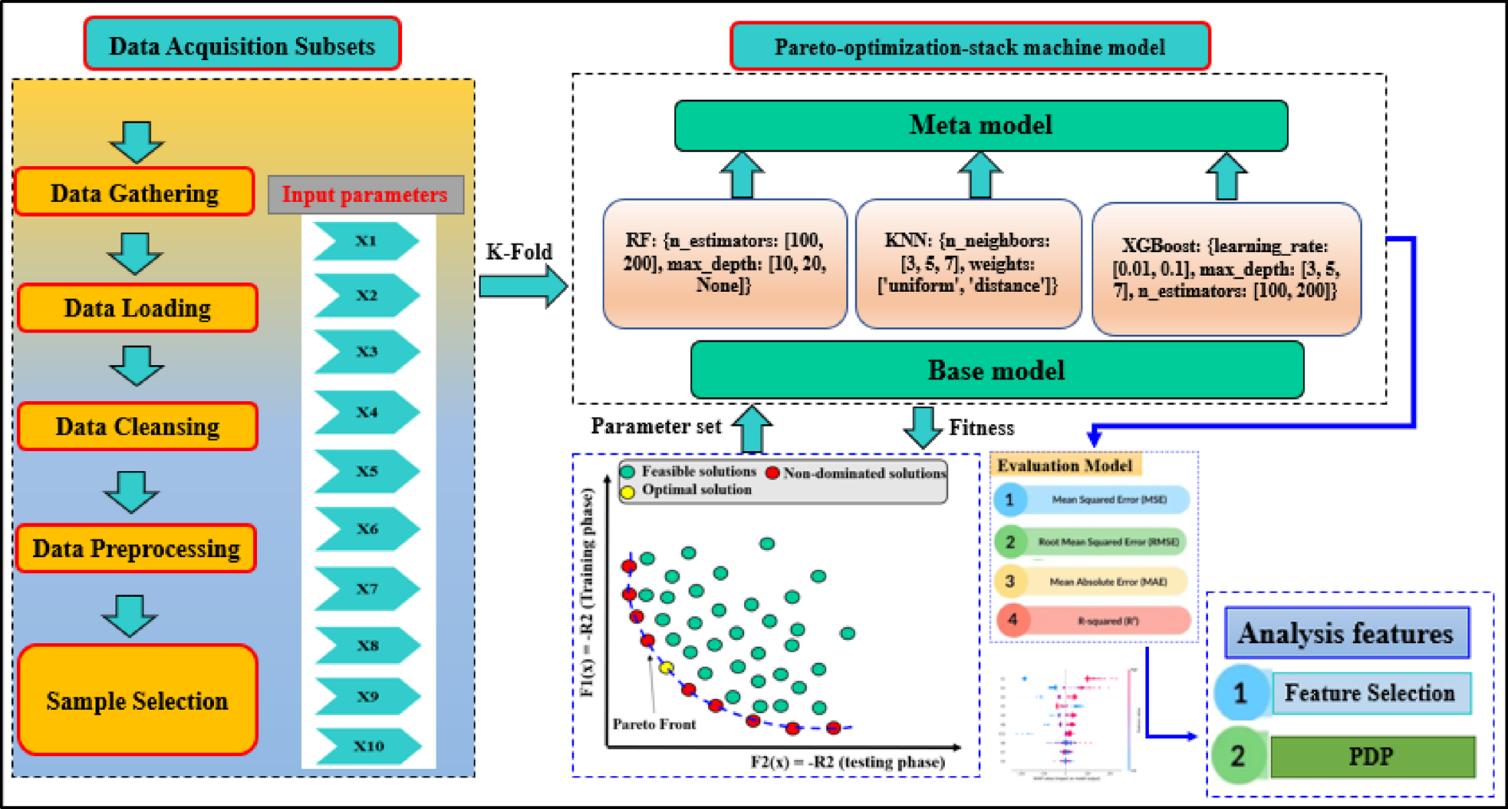
Flowchart of the Pareto-optimized stacking model with RF, KNN, and XGBoost as base learners.

Stacking is an ensemble learning methodology that combines the predictions from a boost of individual models to enhance overall prediction accuracy. Figure 7 shows a schematic representation of the stacking process for regression. For the base model selection, RF, KNN and XGBoost were the base models chosen for the current stacking approach owing to the fact these models provided superior and consistent results and were widely applicable, based on previous studies^21,59,60^; likewise, all three models scored the highest ranked metrics, and all three models gave the highest ranked metrics making them an ideal ensemble model for the task. The thorough evaluation of these five models allowed for an informed choice and subsequently enabled ensemble modeling that is described in the next sections. Specifications of parameter values for the RF, XGB, and KNN models are provided in Table 3.

**Table 3:** Comprehensive parameter details with and without Pareto optimization.

Design variable	Type	Initial value	Range	Pareto optimization
Random forest (base & final estimator)				
n_estimators	Integer	200	[50, 500]	326
max_depth	Integer	10	[3, 30]	27
random_state	Integer	42 (fixed)	–	42 (fixed)
K-nearest neighbors				
n_neighbors	Integer	5	[3, 20]	7
XGBoost				
Learning_rate	Real	0.1	[0.001, 0.5]	0.063
n_estimators	Integer	200	[50, 500]	1000
max_depth	Integer	6	[3, 10]	9
subsample	Real	1.0	[0.5, 1.0]	0.619
colsample_bytree	Real	1.0	[0, 1]	1
lambda	Real	1.0	[0, 1]	0.181
alpha	Real	0.0	[0, 1]	0.326
gamma	Real	0.0	[0, 10]	0.007
min_child_weight	Integer	1	[1, 10]	1

To minimize the systematic biases in data selection and to evaluate the current models appropriately, five-fold cross-validation (CV) was utilized, which is arguably the most established method for evaluating machine learning models, as shown in Fig. 7. In this study, the validation subset used in this process was reserved solely for parameter optimization to avoid overfitting and to ensure the performance without bias. Finally, 4 statistical metrics were used: mean squared error (MSE), root mean squared error (RMSE), mean absolute error (MAE), and the coefficient of determination (R^2^), to assess the performance of the proposed models as detailed in subsequent sections.

#### The multi-objective optimization algorithm

**Fig. 8:**
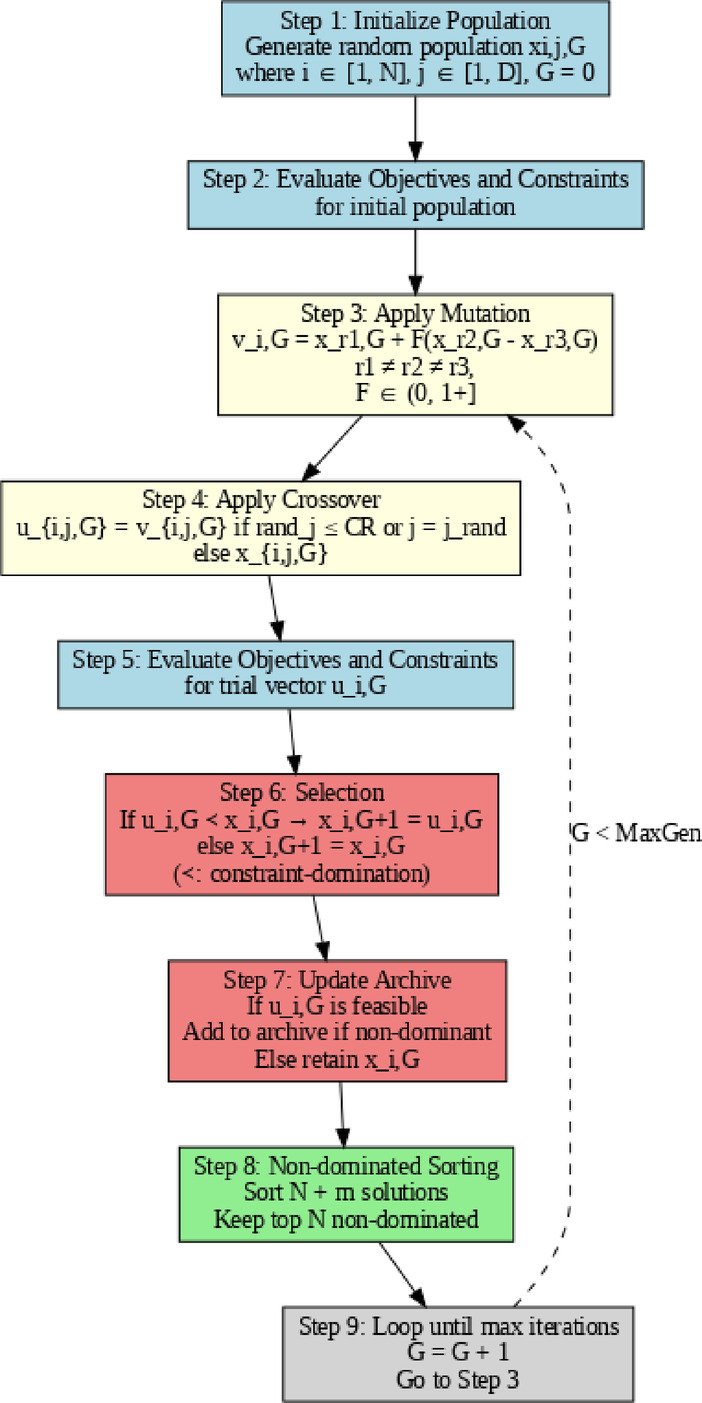
Flowchart of the GDE3-based Pareto optimization process.

The Generalized Differential Evolution Algorithm (GDE3), developed by Kukkonen and Lampinen^61^, is a robust extension of differential evolution for tackling multi-objective optimization problems^62^. The GDE3-based Pareto optimization process is shown as a flowchart in Fig. 8. The algorithm begins with initializing a random, valid population, which is then evaluated for objective functions and constraint functions. The operators of mutation and crossover are applied, resulting in a series of trial vectors. The process of selection employs constraint-dominance to produce a new population. The updated archive of non-dominated feasible solutions is iteratively improved, and the next iteration employs non-dominated sorting to retain the best solutions. The population is iterated until the maximum number of generations has passed. The GDE3 algorithm relies on the following key equations to perform multi-objective optimization with constraint handling:

12 $$\user2{ }{\text{ Mutation}}:\quad {\text{ v}}_{{{\mathrm{i}},{\mathrm{G}}}} = {\mathrm{x}}_{{{\mathrm{r}}1,{\mathrm{G}}}} + {\text{ F}}({\mathrm{x}}_{{{\mathrm{r}}1,{\mathrm{G}}}} + {\mathrm{x}}_{{{\mathrm{r}}1,{\mathrm{G}}}} ),r_{1} \ne r_{2} \ne r_{3} , F \in \left( {0,1 + } \right]$$

13 $${\mathrm{Crossover}}:\quad {\mathrm{u}}_{{{\mathrm{i}},{\mathrm{j}},{\mathrm{G}}}} = \left\{ {\begin{array}{*{20}c} {{\mathrm{v}}_{{{\mathrm{i}},{\mathrm{j}},{\mathrm{G}}}} ,} & {if\, rand_{d} \le CR or j = j_{rand} } \\ {{\mathrm{x}}_{{{\mathrm{r}},{\mathrm{j}},{\mathrm{G}}}} ,} & {otherwise} \\ \end{array} } \right\}$$

14 $${\mathrm{Selection}}: \quad {\mathrm{x}}_{{{\mathrm{i}},{\text{ G}} + 1}} = \left\{ {\begin{array}{*{20}c} {{\mathrm{u}}_{{{\mathrm{i}},{\mathrm{G}}}} , } & {if\, {\mathrm{u}}_{{{\mathrm{i}},{\mathrm{G}}}} \prec {\mathrm{x}}_{{{\mathrm{i}},{\text{ G}}}} } \\ {{\mathrm{x}}_{{{\mathrm{i}},{\mathrm{G}}}} ,} & {otherwise} \\ \end{array} } \right\}$$

where “$\prec$” denotes dominance according to constraints and objective values.

#### Performance indicators for ML validation

The R^2^, MSE, MAE, and RMSE are calculated as presented by Eqs. 15–17, respectively, to evaluate the performance of the proposed algorithms.

- **R**^**2**^ quantifies how well a model fits the data, with values of 0 to 1. A value of 1 demonstrates a perfect fit between the model and the data.
  15 $$R^{2} = 1 - \frac{{\mathop \sum \nolimits_{i} \left( {y_{i} - \hat{y}_{i} } \right)^{2} }}{{\mathop \sum \nolimits_{i} \left( {y_{i} - \overline{y}_{i} } \right)^{2} }}$$
- **MSE** calculates the average of the squared differences between predicted and actual values, giving more weight to larger errors.
  16 $$MSE = \frac{1}{n}\mathop \sum \limits_{i = 1}^{n} \left( {y_{i} - \hat{y}_{i} } \right)^{2}$$
- **RMSE** is a statistical metric that represents the average magnitude of errors.
  17 $$RMSE = \sqrt {\frac{1}{n}\sum\nolimits_{i = 1}^{n} {\left( {y_{i} - \hat{y}_{i} } \right)}^{2} }$$
- **MAE** quantifies the average discrepancy between predicted and experimental datasets.
  18 $$MAE = \frac{1}{n}\sum\nolimits_{i = 1}^{n} {\left| {y_{i} - \hat{y}_{i} } \right|}$$
  where y_i_, $\hat{y}_{i}$ and $\overline{y}_{i}$ represent the true, predicted, and average CS results of the FC, respectively.

## Results and discussions

### Model performance evaluation

#### Base model performance

**Fig. 9:**
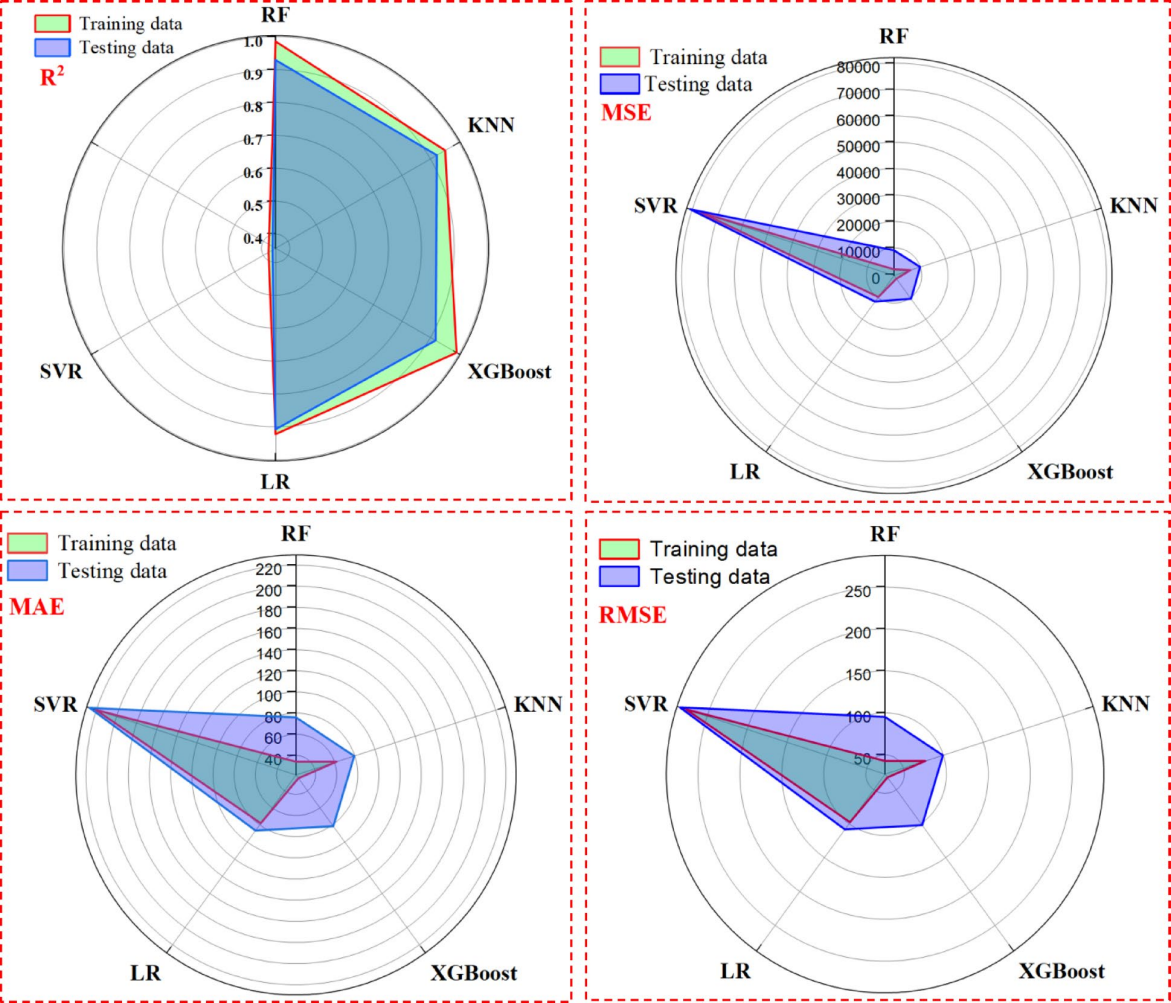
Circular radar diagram indicating the five-individual algorithm performance.

The radar diagram shows the performance of 5 key base models: RF, LR, SVR, XGBoost, and KNN. These models were assessed, and to measure their performance, MSE, RMSE, MAE, and R^2^ were recorded for training and testing datasets. This multi-staged analysis demonstrates the performance of each model, its strengths, and weaknesses. The best model was RF, which had the lowest MSE, RMSE, and MAE and the highest R^2^, respectively, when looking at test data. Therefore, the models’ ranking is 1, RF; 2, KNN; 3, XGBoost; 4, LR; 5, SVR. As shown in Table 4 and Fig. 9, the average values from 20 independent runs indicate RF’s strong diagnostic capability, with R^2^ values of approximately 0.9854 and 0.9292 for training and testing, respectively, alongside the smallest MSE, RMSE, and MAE among the models. However, Fig. 9 demonstrates a significant gap between training and testing data for RF, XGBoost, and KNN methods, likely due to overfitting from default parameter settings. A new framework was used that utilized predictions from multiple models to accurately predict outcomes while limiting the chances of overfitting. Also, hyperparameters were tuned intensely and produced some very good improvements to the prediction performance. Reflecting upon these findings shows how important it is to tune parameters and new model frameworks to achieve good prediction performance. In the following examination, RF, KNN, and XGBoost were the three models selected for base models in the stacking ensemble model because of their strong performance relative to the other models. A full evaluation of the ensemble approach was then considered on a combination of: (i) the individual RF model, (ii) the individual KNN model, (iii) the individual XGBoost model, (iv) stacking RF, KNN, and XGBoost models (stacking ensemble), and (v) stacking RF, KNN, and XGBoost models with Pareto optimization.

**Table 4:** Performance evaluation of the individual base models.

Model	MSE		RMSE		MAE		R^2^		Ranking
	Training	Testing	Training	Testing	Training	Testing	Training	Testing	
RF	1806.13	9041.07	42.49	95.08	33.9	75.86	0.9854	0.9292	1
KNN	5988.82	9880.06	77.38	99.39	61.74	79.3	0.9515	0.9226	2
XGBoost	1009.68	10436.21	31.77	102.15	25.35	81.5	0.9918	0.9183	3
LR	9587.35	11761.91	97.91	108.45	78.12	86.53	0.9223	0.9079	4
SVR	76356.69	80681.18	276.32	284.04	220.47	226.63	0.3812	0.368	5

**Fig. 10:**
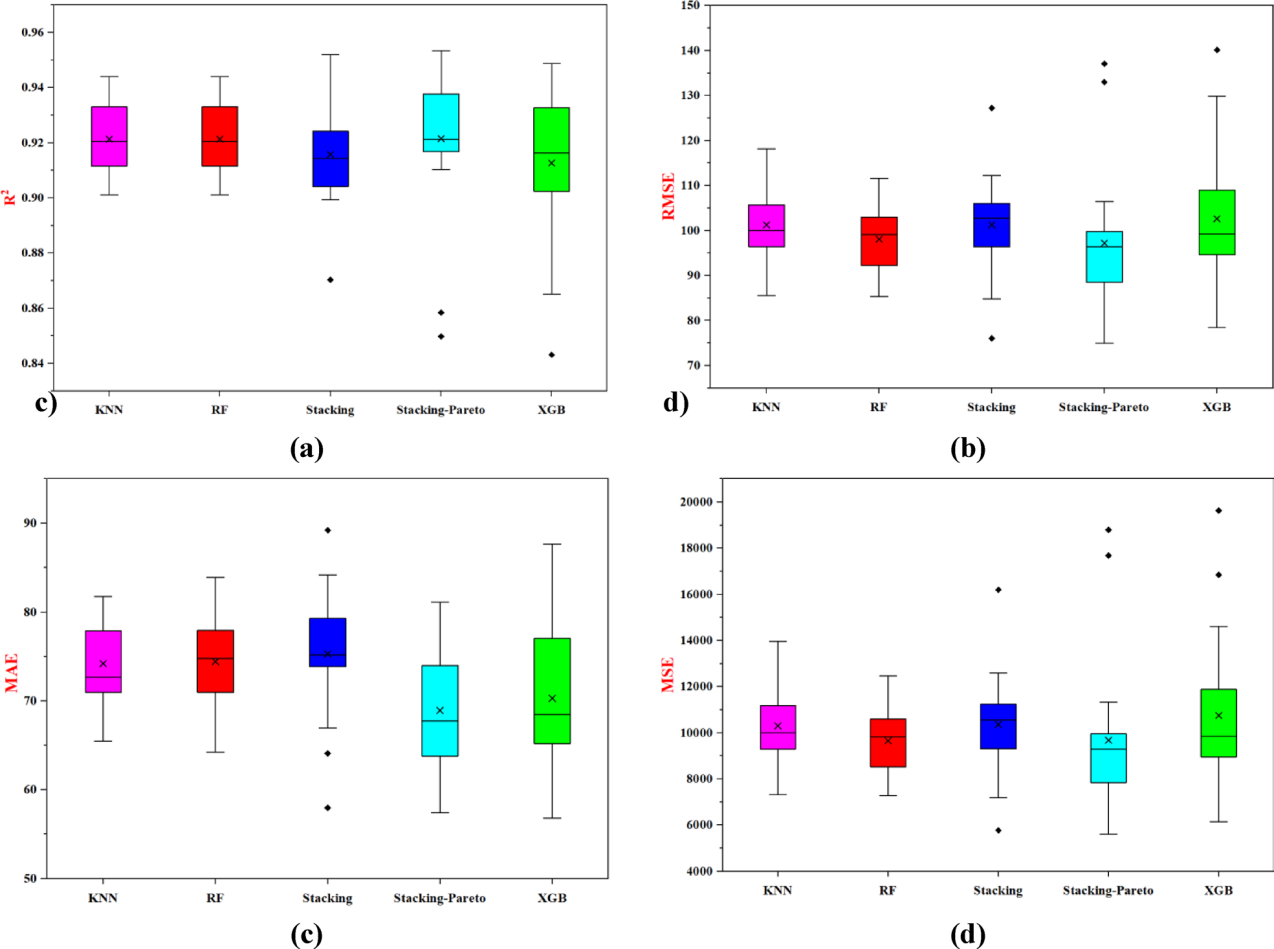
Comparative performance of stacking models on testing phase: (**a**) R^2^, (**b**) RMSE, (**c**) MAE, (**d**) MSE.

The evaluation results, summarized for the testing data in Fig. 10 and detailed in Table 5, indicate the models’ accuracy in capturing the complex characteristics of pile-bearing capacity in the training and testing datasets. The box plot shows the Pareto optimization model being the highest performer with a higher correlation coefficient than the other models across the training and testing datasets. In addition, this result confirms that this method best fits the real-world data, illustrating the effectiveness of stacking and Pareto optimization. Table 5 also shows that the stacking ensemble model surpassed the metrics in the standalone RF model, in addition to one performance metric. Overall, these results have strong implications for pile foundation design. The Pareto optimization model’s strong correlation with observed data makes it an optimal choice for accurate predictions of driven pile bearing capacity, positively impacting the accuracy of foundation design. Stacking ensemble models has demonstrated a high ability to identify minor behaviors that were not captured with the single models. Taken as a whole, these findings demonstrate that ensemble modeling techniques are better than single models and allow the use of Pareto optimization and stacking, which would inspire new models to model PBC and provide a sound basis for ensuring safe and efficient pile construction. The current results are consistent with previous studies, particularly^8,37,63,64^, where they developed an interpretable stacked ensemble model optimized through Pareto multi-objective optimization to accurately model the load-displacement behavior of precast prestressed centrifugal concrete piles.

**Table 5:** Performance evaluation of the stacking ensemble and Pareto-optimized models.

Model	MSE		MAE		RMSE		R^2^		Ranking
	Training	Testing	Training	Testing	Training	Testing	Training	Testing	
RF	1806.13	9041.07	33.9	75.86	42.49	95.08	0.9854	0.9292	3
KNN	5988.82	9880.06	61.74	79.3	77.38	99.39	0.9515	0.9226	4
XGBoost	1009.68	10436.21	25.35	81.5	31.77	102.15	0.9918	0.9183	5
Stacking ensemble	3715.15	8491.15	53.67	75.28	60.95	92.14	0.9699	0.9335	2
Pareto-optimization	5268.26	7032.20	55.78	68.96	72.58	83.85	0.9573	0.9471	1

**Fig. 11:**
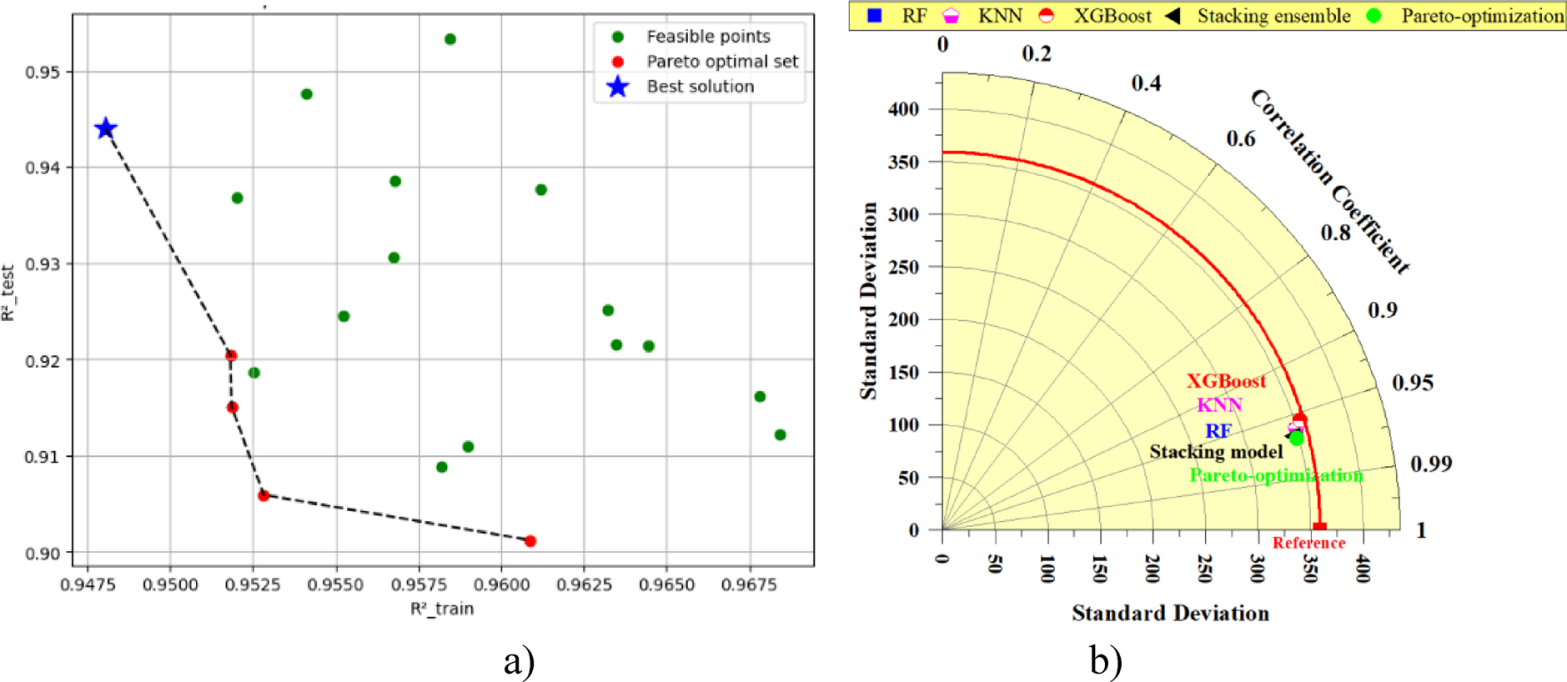
(**a**) Pareto optimization of the parameters stacked model and (**b**) Taylor diagram of the development of the best individual and stacked model with Pareto optimization.

Clearly, the application of Pareto multi-objective optimization significantly improved the performance of the stacking model. The enhancements to the model can be clearly seen in Fig. 11a, which shows the behavior of the model through the Pareto optimization technique. The Pareto optimization approach definitively improved the predictive performance of the model on the testing subset to that of the training subset performance. In Fig. 11a, the green points show possible solutions, the black dotted line shows the Pareto front set, and the blue star indicates the optimal solution, with an R^2^ of about 0.9471 on the test set to minimize the negative R^2^ and to try to avoid overfitting. This confirms that the best combination of factors from Table 2 for this data was used to forecast the PBC for driven piles. Additionally, Fig. 11b offers a more direct comparison of model performance through a Taylor plot, which plots the standard deviation against the correlation coefficient. The Pareto-optimization model (green) rests closest to the ideal reference line, suggesting a more consistent and overall more accurate model than still stacking ensemble (black), XGBoost, KNN, and RF models. Regardless of the aggregation information, there is a distinct advantage for the Pareto-optimization model over every other model, allowing this to be visualized. This further substantiates that Pareto optimization was completed successfully, aligns with the metrics and values from each model displayed in Fig. 10 and Table 5, and allows for the conclusion to be made that Pareto optimization is the appropriate method for making robust predictions of pile bearing capacities.

**Fig. 12:**
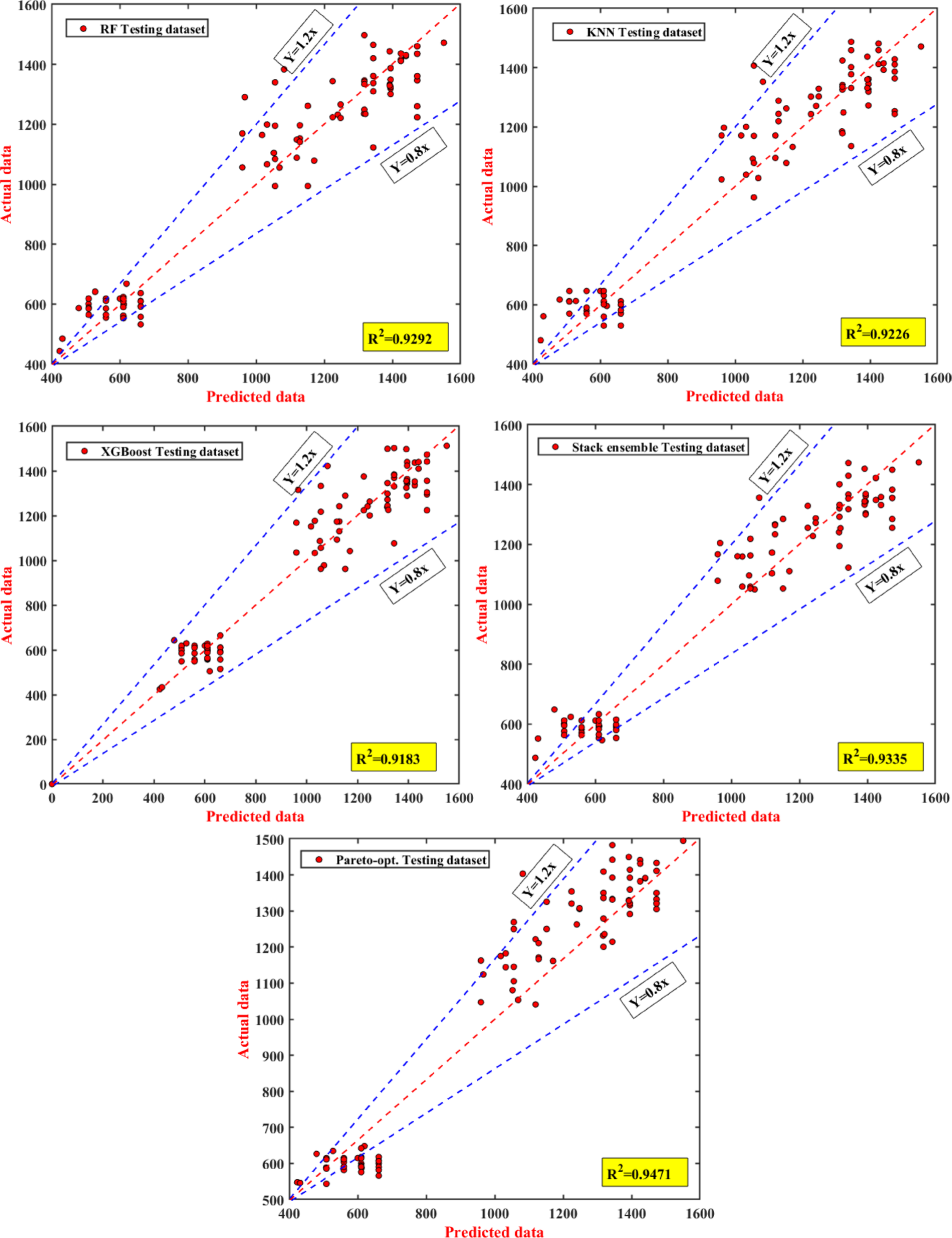
The performance of the best individual and stacked model with Pareto optimization for the testing dataset.

Figure 12 provides a detailed examination of the predictive performance of the individual models and the stacking of models, including RF, KNN, XGBoost, as well as the overall ensemble, and the Pareto-optimized model through the scatter plots comparing the predicted and actual PBC values in the testing data set. The R^2^ values can serve as a suitable metric of model accuracy, where RF exhibited an R^2^ of 0.8292, KNN had an R^2^ of 0.9276, and XGBoost had an R^2^ of 0.9163, demonstrating variance in model performance. The stacking ensemble built upon these values, demonstrating an R^2^ of 0.9335, showing the potential of applying multiple base models as well. The Pareto-optimized model presented a further improvement, with an R^2^ of 0.9471, demonstrating its impressive ability to join predicted values with actual data points, generating lessened prediction error. In other words, its improved performance reflects its obvious capacity to predict when built from the nested sub-ensemble models of the stacking ensemble. The scatter points that were used to determine testing performance closely match the fitted line, exhibiting a strong correlation between predicted and actual PBC values, which indicates that this model was able to generalize learned data and subsequently present a low risk of overfitting or underfitting. A low amount of variability around the fitted line continues to indicate that this model is capable of consistently and accurately predicting performance across a varying PBC. However, as shown in Fig. 12, there was a slight deviation of points from the fitted line at the higher displacements, which could indicate that the testing dataset may not have been extensive enough to include extremes of load conditions. The progressive improvement across models demonstrates the importance of advanced optimization methods in geotechnical engineering. Enhancing our ability for accurate PBC predictions provides the basis for safer and more reliable foundation systems.

In summary, the regression plot in Fig. 12 provides compelling proof of the stacking model’s efficacy when integrated with Pareto optimization. Its impressive determination coefficients and consistent predictive accuracy affirm its ability to reliably model the PBC behavior. The outstanding success of this model can be traced to thoughtful strategic decisions, notably the innovative decision to divide the pile into ten distinct segments and incorporate effective overburden pressure across these divisions, forming a cornerstone of this research. This breakthrough carries significant implications for geotechnical engineering, delivering a dependable tool that can substantially improve the design and construction of foundation systems, especially those dependent on driven piles.

**Fig. 13:**
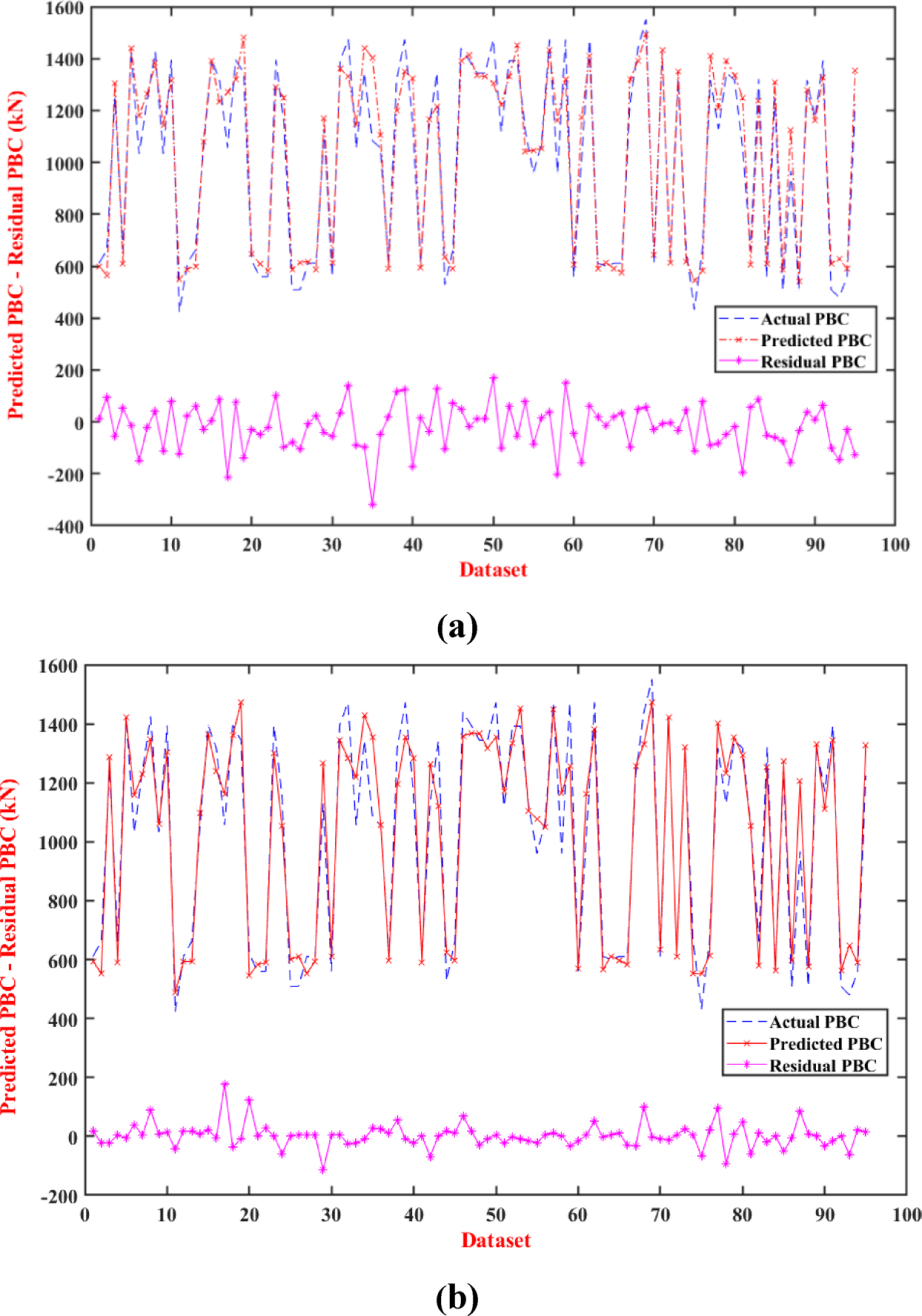
Prediction results of stacked ensemble model, (**a**) with and (**b**) without Pareto optimization for testing dataset.

Fig. 13 presents a comparative analysis of the prediction results for the stacking model, with and without Pareto optimization, applied to the testing dataset, offering valuable insights into the model’s performance in predicting PBC. Fig. 13a illustrates the outcomes for the stacking ensemble model enhanced with Pareto optimization, where the predicted PBC values (red line) closely track the actual PBC values (blue line) across the dataset. This alignment is indicative of the model’s high predictive accuracy, with minimal deviation, reflecting the effectiveness of Pareto multi-objective optimization in refining the model’s ability to generalize and capture complex PBC behaviors. In contrast, Fig. 13b displays the results for the stacking ensemble model without Pareto optimization, where the predicted PBC values exhibit greater fluctuations and larger discrepancies from the actual PBC values. This increased variability suggests a reduced capacity to accurately model the dataset, demonstrating the limitations of the unoptimized approach in handling the intricate dynamics of pile behavior. The visual evidence in Fig. 13 complements the statistical findings from previous analyses, such as the R^2^ values in Fig. 12, where the Pareto-optimized model achieved an R^2^ of 0.9471, compared to the lower performance of individual models and the unoptimized stacking ensemble. The smoother and more consistent predictions in Fig. 13a reveal the critical role of Pareto optimization in enhancing model stability and precision, particularly for testing data that may include diverse or challenging conditions.

### Model explanation

#### SHAP summary plot

**Fig. 14:**
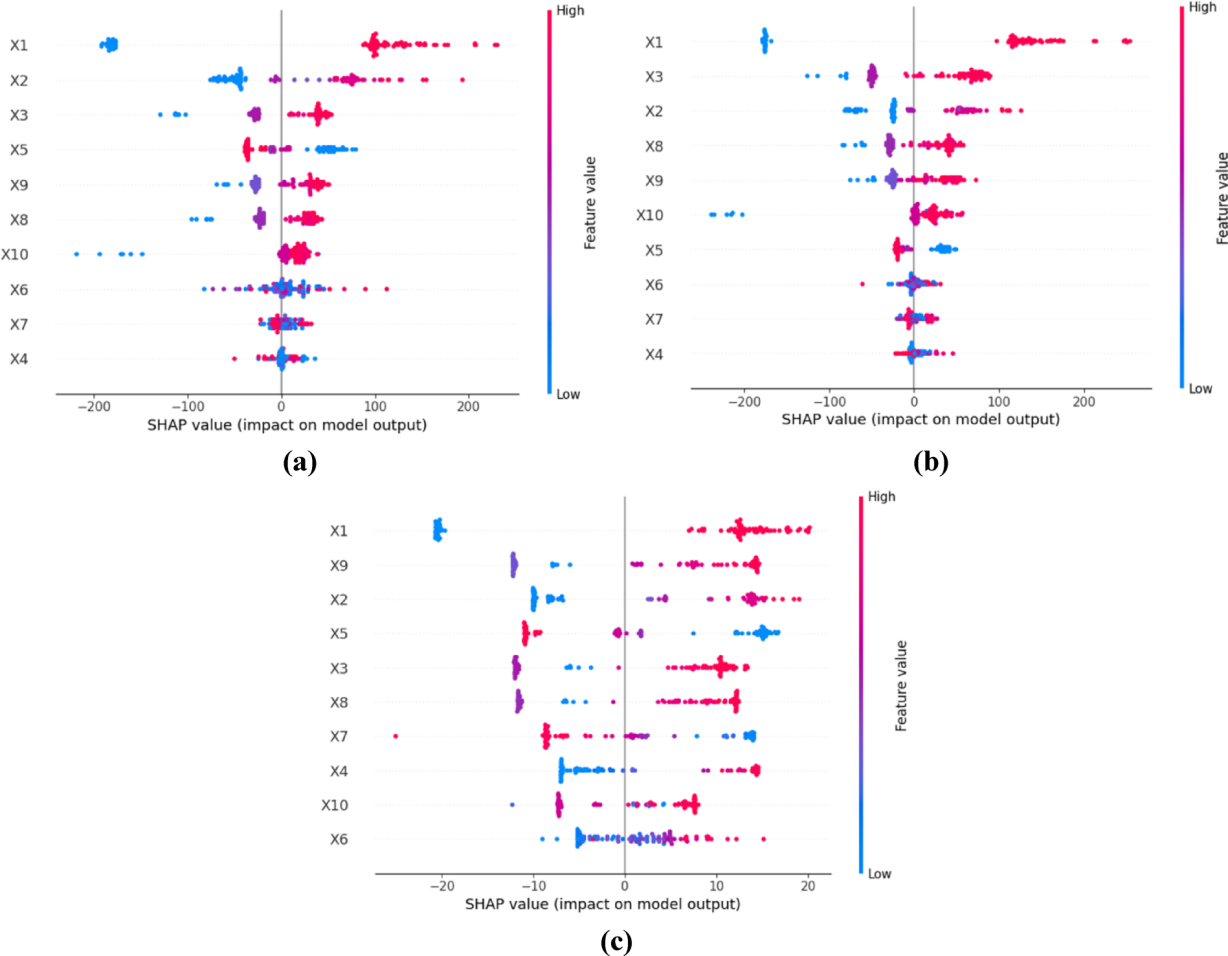
Influence of features: SHAP summary plot (**a**) RF, (**b**) Stack ensemble, and (**c**) stacked model with Pareto optimization.

SHAP is used to determine the relative importance of independent variables in predicting the outcome, where a higher relative importance indicates a greater impact of a variable on the prediction of the model. The SHAP summary plots in Fig. 14 depict how the features influence model predictions. In this case, there are three model configurations: (a) RF, (b) stacking ensemble, and (c) stacking ensemble with Pareto optimization. Each plot depicts SHAP values for a feature for a prediction as points. The horizontal axes indicate the direction and size of the impact feature on the prediction, so positive values increase the prediction, negative values decrease the prediction, and the color gradient (*blue* = *low, red* = *high*) indicates the size of the feature. Considering the RF model (Fig. 14a), features X1, X2, X3, X5 & X9 have the largest SHAP ranges, and had the largest impact in model predictions. When X1 (pile diameter) is high, predictions are quite a bit higher, and conversely, when X2 is high, predictions tend to be quite a bit lower. The SHAP values for these two features were about ± 200, indicating these features contributed to a great deal of variability in predictions. Though the stacking ensemble (Fig. 14b) has a similar rank-order of feature importance in that X1, X3, X2, X8, and X9 are the most important, it appears that the SHAP values are more widely separated, indicating that ensembling may mitigate the extreme biases of RF and have a similar degree of spread around ± 200. Particularly, X1, X2, and X3 are still the most important features in Fig. 14a, with the color from blue to red denoting the range of the feature values. In contrast, it appears that the Pareto-optimized stacking model (Fig. 14c) has a reduced SHAP magnitude of approximately ± 20, indicating that optimization results in greater model regularization, reduced influence from the leading features, and improved stability.

It was observed that X1, X9, X2, and later X5 influence the model, but with a more uniform influence across the observations. Across models, X1 is arguably the most critical feature, while Pareto optimization reduced extreme influences of features to the extent that a more equitable interaction between accuracy and robustness in the model can be concluded. Furthermore, X9 (Average SPT-N along shaft) is the most significant for Fig. 14c compared to 14a and 14b, highlighting that its importance can vary depending on the modeling methodology adopted.

#### SHAP feature importance

**Fig. 15:**
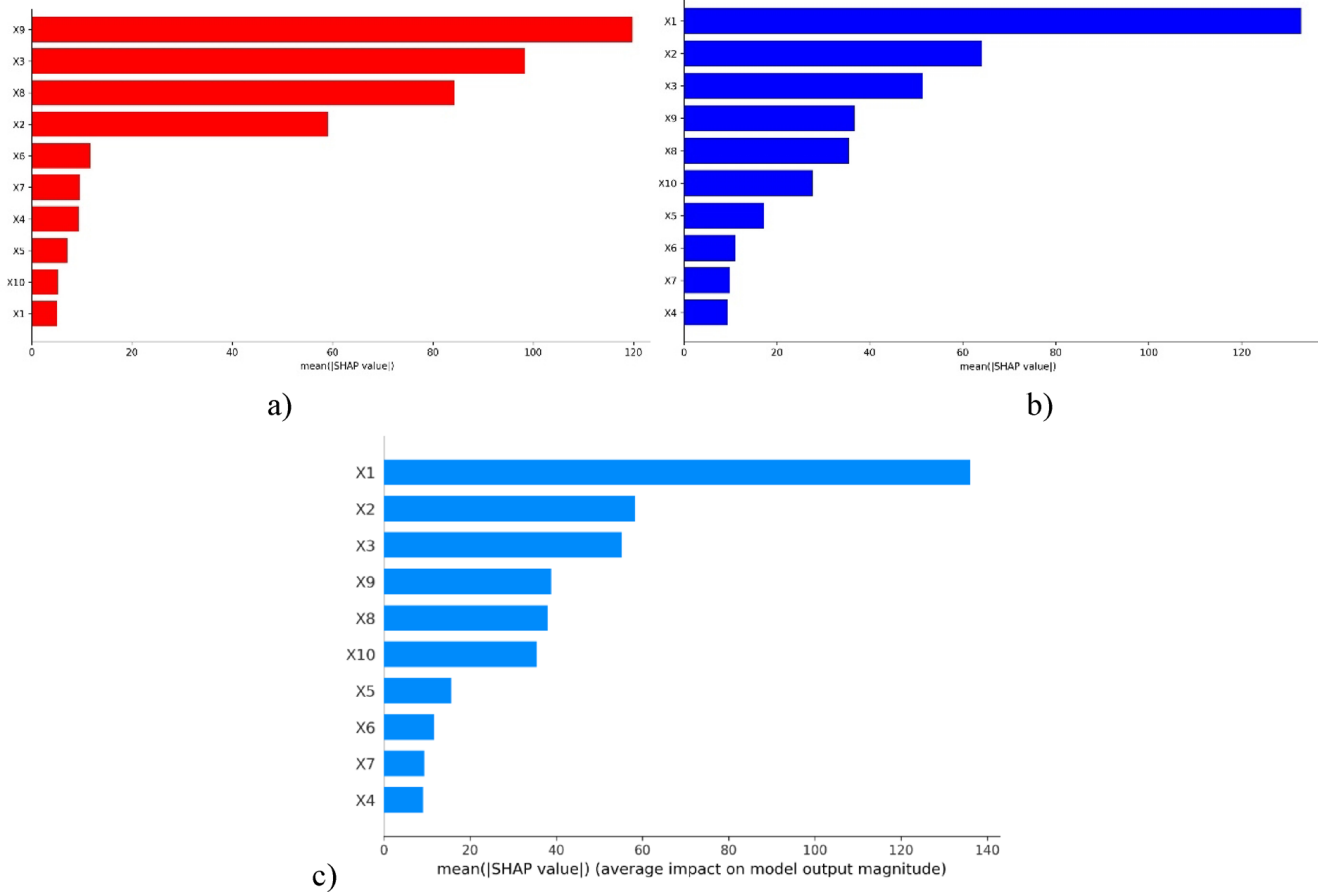
Influence of features: SHAP summary plot (**a**) RF, (**b**) Stack ensemble, and (**c**) stacked model with Pareto optimization.

In this study, the input features on the model outcome are evaluated with mean absolute SHAP values, which indicate that larger SHAP values contribute more influence when predicting PBC. SHAP was applied to the RF, the stacking ensemble, and the Pareto optimization models. The average SHAP values of all the features for the models are shown in Fig. 15. The findings of the RF model, shown in Fig. 15a, demonstrate that the sample features X9, X3, X8, and X2 exhibited the highest SHAP values, suggesting these features were the most influential to the estimation of PBC. The remaining features had lower SHAP values, indicating a relatively lower contribution in predicting PBC. Concerning the stacking ensemble and Pareto optimization models (see Fig. 15 b, c, respectively), the results suggest X1 (pile diameter) is the most important feature (highest SHAP value), while nearly all other features show low SHAP values, suggesting they have low impact on the PBC prediction. Among the features, X1 (pile diameter) consistently shows the highest impact on the variation in PBC in either of the stack models. The RF model appears to show a more distinct separation in terms of feature importance, with X9, X3, X8, and X2 being observed, whereas the stacking with or without the Pareto optimization model emphasizes a more uniform contribution across features, with X1 still being the biggest contributor. The analysis clearly shows the importance of X1 in PBC estimation, evident in the high SHAP values of both modeling techniques.

#### Sensitivity analysis and parametric analysis

The accuracy and reliability of the ML-based prediction models for PBC were further checked by conducting sensitivity analysis (SA) and parametric testing. The sensitivity index (SI) ranks the independent variables by importance and allows us to assess the importance of that variable concerning the model predictions made. For this study, a sensitivity analysis was performed by using Eq. (19)^65^ as defined:

19 $$SI = \frac{{\mathop \sum \nolimits_{i = 1}^{n} \left( {P_{i}^{exp} \times PBC_{i}^{Pred} } \right)}}{{\sqrt {\mathop \sum \nolimits_{i = 1}^{n} \left( {P_{i}^{exp} } \right)^{2} \times } \mathop \sum \nolimits_{i = 1}^{n} \left( {PBC_{i}^{Pred} } \right)^{2} }}$$

where $P_{i}^{exp}$ represents the experimental input parameter, and ${ }PBC_{i}^{Pred} { }$ denotes the predicted PBC. The SI value ranges from 0 to 1, with a value approaching 0 indicating minimal significance and a value nearing 1 reflecting a strong sensitivity of the independent parameter to the predicted PBC.

**Fig. 16:**
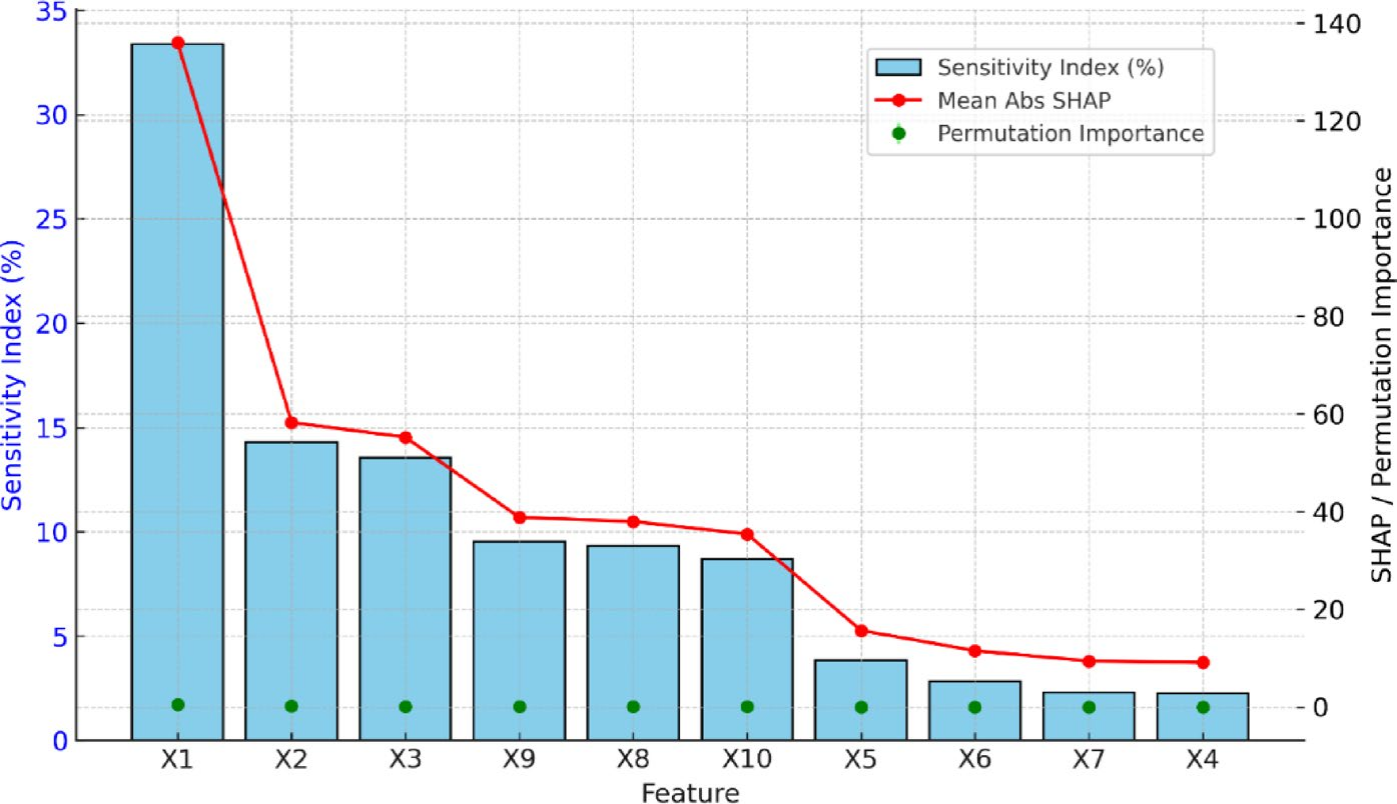
Comparison of feature importance measures for predicting PBC using SI (%), Mean absolute SHAP values, and permutation importance.

Fig. 16 shows the results from the sensitivity analysis of the Pareto optimization model with both quantitative and qualitative variables. There are many different curves shown that are traced out using the input variable and their respective SI values to help visualize their influence on the predicted PBC. The largest SI value of 0.35 can be seen in the curve for X1 (pile diameter), meaning that pile diameter is the most sensitive parameter that has a significant impact on the PBC value since it is directly responsible for the distribution of loads and soil resistance. The curve for X4 has the smallest SI value, meaning that this parameter is the least influential. The other curves presented in Fig. 16, which represent parameters such as X3 and X5-X10, show intermediate SI values between 0 and 0.15, indicating moderate influence. These results confirm an indication that model optimization and design should take advantage of prioritizing X1 and X2. So, based on the feature importance analysis of predicting PBC through the Pareto optimization model, the SHAP value, and permutation importance produced similar findings across the three different methods: sensitivity index, SHAP values, and permutation importance. In terms of inputs, X1 stands as the dominant factor; it not only presents the highest sensitivity with the highest percentage, but also has the largest SHAP value, and permutation importance of all variables gives X1 a leading role overall to control predictions of the model. Although X2 and X3 follow in a role of significant contributor, but considerably lower than X1, the variables X4, X6, and X7 have a very low importance across all methods; meaning it is not likely to contribute much to the prediction accuracy of the interest variable. Given the significance of the agreement arrived at, three techniques provide a lot of confidence in conclusions and can only confirm that PBC was attained through a few parameters. X1 presumably dominates capacity prediction, followed by X2 and X3.

**Fig. 17:**
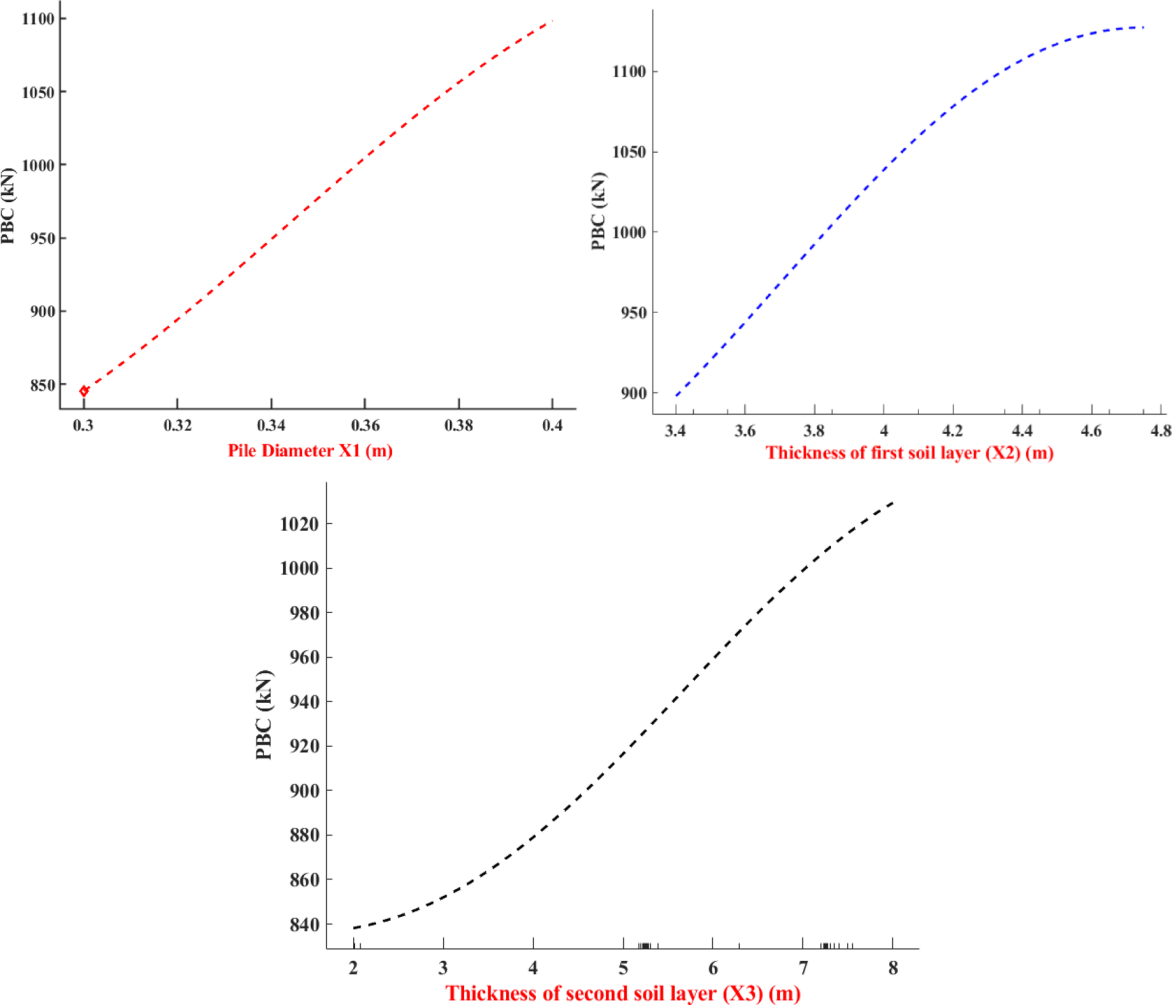
Effect of high sensitivity parameters on predicted PBC: (**a**) X1; (**b**) X2; and (**c**) X3.

The parametric analysis was conducted to confirm whether the proposed prediction models represent the underlying physical behavior rather than statistical correlations. The “one-at-a-time” (OAT) procedure was used to individually vary one parameter, while keeping the other input parameters at its baseline values and assess the parameter’s effect on the predicted PBC (Fig. 17). During the analysis, the pile diameter (X1), thickness of first soil layer (X2), and thickness of second soil layer (X3) were determined to be highly sensitive parameters. The PBC increased consistently with pile diameter and soil layer thickness in accordance with basic geotechnical principles, confirming the physical significance of the prediction model, and behavior consistent with the feature importance analysis previously conducted.

#### Effect of the combination of PBC parameters using the Pareto optimization model

**Fig. 18:**
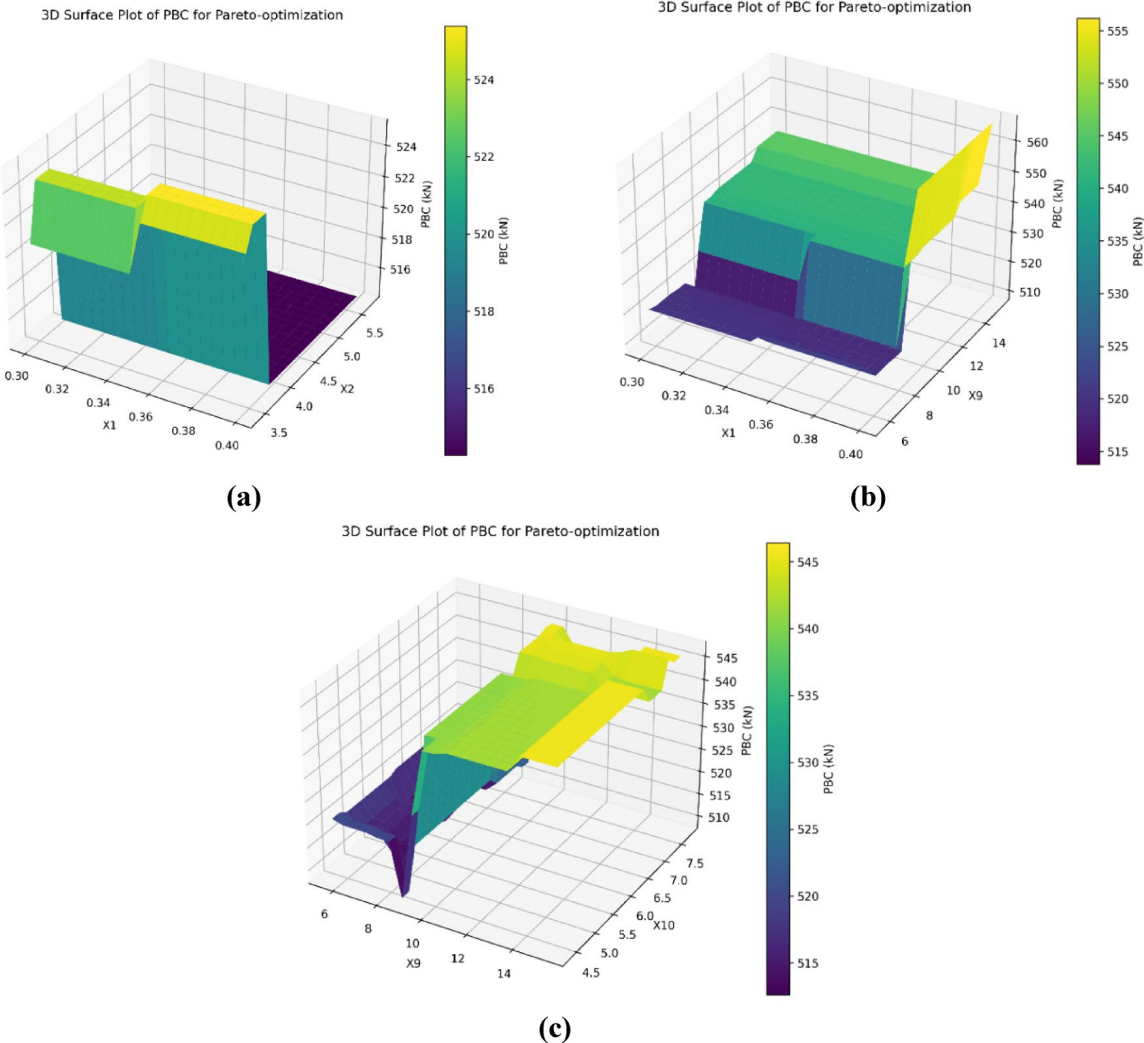
Influence of combined variable parameters on the PBC, (**a**) X1 vs.· X2, (**b**) X1 vs. X9, (**c**) X9 vs. X10 using the Pareto optimization model.

A full understanding of the individual influences of the parameters of each of the estimated PBC parameters will help to optimize the design of pile foundations. In practice, the PBC parameters have complex and sometimes non-linear interactions, which may produce good synergistic operation or sometimes inhibit overall performance. The independent parameters could also lead to nonlinear responses; therefore, it was important to assess both the individual and concurrent effects of the parameters at PBC. The analysis of selected pairs of experimental factors will collectively influence PBC parameters according to the results of Pareto optimization model, presented in Fig. 18. The large influence of pile diameter (X1) and thickness of the first soil layer receiving the pile (m) on the PBC is demonstrated in Fig. 18a. At smaller pile diameters (<0.35 m) and small thicknesses of the first soil layer (<4.0 m), the PBC decreases nearly 40% in some cases, as low as 516 kN in the most limiting condition, and clearly demonstrates the potential for two critical parameters embedded in the pile, and their role in the fully embedded layer, to significantly change PBC. As the pile diameter increases beyond the 0.35 m diameter, and the depth of the first soil layer is greater than 4.0 m in thickness, the PBC rapidly improves. This indicates that there is not only a positive enhancement to PBC from X1, but also from the thickness of the first soil layer that improves the load distribution and ultimately increases soil resistance.

Fig. 18b evaluates the joint effect of X1 and average SPT-N along the shaft (X9) on PBC. At smaller pile diameters (<0.35 m) achieved through an SPT-N, the shaft PBC stabilizes at approximately 520 kN. Moreover, as the pile diameter increases and SPT-N along the shaft is greater than 10, PBC continues to increase. It appears that larger diameter piles, in soils with higher shaft resistance, can significantly increase bearing capacity. This increase in bearing capacity is due to added frictional resistance associated with increased appreciation of friction resistance along the shaft and the role this plays in PBC. Fig. 18c examines the interaction of average SPT-N at the tip (X10) and X9 on PBC. In general, if the SPT-N at the pile tip is low (<5) and the average SPT-N along the shaft is less than 10, the PBC will be below 500 kN. The results of PBC increased rapidly after the SPT-N at the tip reached 7, and the average SPT-N along the shaft reached 10. It should be noted that a strong soil layer at the pile tip (SPT-N >7) and sufficient shaft resistance (SPT-N >10) improve end-bearing capacity and are substantial contributors to PBC. Overall, these findings demonstrate the necessity to assess the concurrent effects of PBC parameters. A proper pile foundation considers good integrated site and structural design to maximize PBC.

### Residual analysis of predicted PBC

**Fig. 19:**
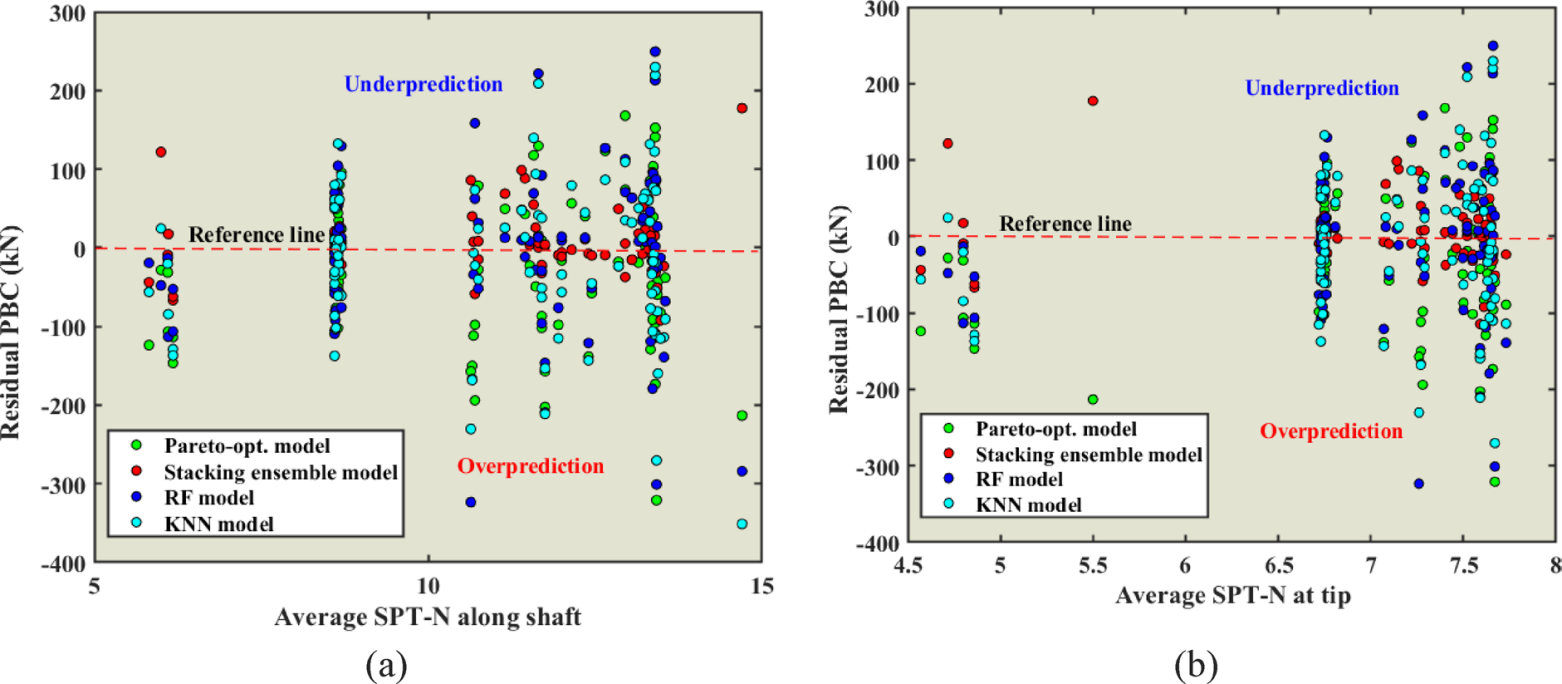
Residuals of predicted PBC versus average SPT-N along the shaft (**a**) and at the pile tip (**b**) for Pareto-optimized, stacking ensemble, RF, and KNN models.

Residual distributions of predicted PBC versus the mean SPT-N values along the shaft (X9) and at the tip of the pile for each model (X10) using Pareto optimized, stacking ensemble, RF, and KNN models are shown in Fig. 19. The red dashed line in the figures, represents the reference (zero-residual) level of prediction, with predictions above representing underestimation and predictions below representing overestimation. In general, the Pareto optimization and stacking ensemble showed the best ability to predict reliability. Residuals were more tightly grouped around the reference line and had fewer large residuals when compared to RF and KNN. The individual models had a wider scatter of residuals, with individual models showing values as extreme as ±300 kN in some cases, indicating large errors. In addition, the residuals for SPT-N at the pile tip had a greater spread than the residuals along the pile shaft. This was especially true for higher SPT-N values. This suggests that capture of tip resistance is difficult, mainly because of soil variability and end bearing in estimates of capacity. Overall, these results suggest that while advanced ensemble and optimization approaches clearly demonstrate improved reliability of prediction, work to reduce outliers and improve robustness of prediction, especially for tip resistance, continues to be necessary.

## Limitations

Despite the promising predictive performance, this study has several limitations. The dataset, while comprehensive with 472 SLT records, is geographically constrained to Ha Nam province, Vietnam, representing predominantly soft to medium alluvial deposits typical of the Mekong Delta region. This may limit direct applicability to regions with significantly different soil profiles, geological conditions, or pile installation practices.

The input features primarily rely on SPT-N values and geometric parameters (*including pile diameter and soil layer thicknesses X₂–X₄ derived from borehole logs*). Several influential parameters were not considered, such as detailed pile installation effects, long-term soil-pile interaction phenomena, and time-dependent capacity changes beyond the short-term resting period. The model’s performance in predicting outlier or extreme cases within the dataset range also shows residual scatter, as indicated by residual analysis (Fig. 19). In very soft or complex soils, complementary tests such as CPT or Shelby tube sampling may be needed to better characterize weak layers.

A key practical limitation is the strong dependence on detailed in-situ testing data, particularly precise soil layer thicknesses (X₂–X₄). Borehole spacing is often limited in real projects, stratigraphic interpretation involves uncertainty, and high-resolution layer information may not be available at the design stage. Given the substantial influence of these parameters on predicted PBC, as demonstrated by SHAP feature importance and parametric studies, missing or uncertain inputs could lead to increased prediction errors, reduced model reliability, or biased estimates in heterogeneous stratigraphy. In such cases, the framework should be applied cautiously and supplemented with conservative engineering judgment, additional site-specific investigations, or conventional empirical methods. Future work should aim to incorporate more diverse datasets, additional geotechnical parameters, and temporal factors to enhance model robustness and generalizability.

## Conclusions

This paper investigated the predictive performance of five ML models (RF, KNN, XGBoost, LR, and SVR) for estimating the PBC of driven piles. A Pareto-optimized stacking ensemble model was developed and validated on a large dataset of 472 static load tests, with SHAP and PDP used to enhance interpretability. The key findings of this study can be summarized as follows:

1. Among the five proposed ML models, RF exhibited the strongest predictive capability, achieving an average test R^2^ of 0.9292, which was 11.3% higher than KNN (0.8355) and 14.7% higher than XGBoost (0.8102). RF also recorded the lowest MSE, RMSE, and MAE, demonstrating its superior diagnostic ability.
2. The stacking ensemble framework demonstrated clear advantages over individual models. Without optimization, the ensemble increased R^2^ from 92.92% (RF) to 93.35%, while Pareto optimization further improved R^2^ by 2.79%. On the test set, the Pareto-optimized model achieved an MSE of 7032.20, representing a 22.2% reduction relative to RF (9041.07), and lowered MAE by 9.1%, highlighting its enhanced generalization ability.
3. Feature importance and sensitivity analyses consistently identified pile diameter (X1) as the dominant predictor of PBC. Its sensitivity index (0.35) was over 70% higher than other variables (X3, X5–X10, SI ≤ 0.20). SHAP values further showed X1 contributed 40–50% of the total importance, while X9 (average SPT-N along shaft) became nearly five times more influential in the Pareto-optimized model compared to RF, demonstrating the role of soil properties in model robustness.
4. Residual analysis confirmed the reliability of the Pareto-optimized stacking model. Scatter was reduced by up to 50% compared with RF and KNN, with predictions remaining within ± 100 kN of observed values, while RF and KNN produced extreme errors up to ± 300 kN. This robustness highlights the ensemble’s potential for safe and efficient PBC prediction.

In the end, our study fills a significant gap in the literature by showing that ensemble learning with Pareto optimization not only improves prediction accuracy but also guarantees interpretability through SHAP and PDP. The results give geotechnical engineers a trustworthy tool for decision-making, allowing for more precise foundation design and lowering uncertainty in pile construction. To improve model generalization, future research could expand this framework by adding deep learning architectures, more soil-geotechnical factors, and cross-regional transfer learning.

## Supplementary Information

Below is the link to the electronic supplementary material.

Supplementary Material 1

Supplementary Material 2

## Data Availability

The raw data is submitted in supplementary materials file.
