# Supplementary material for: Pareto-optimized stacked ensemble machine learning framework for predicting bearing capacity of driven piles from static load test data
Source: Sci Rep. 2026 Apr 2;16:11360. doi: 10.1038/s41598-026-43660-z (PMC13049102; doi:10.1038/s41598-026-43660-z)
Supplement: Supplementary file 1 — Supplementary Material 1 [file 41598_2026_43660_MOESM1_ESM.docx]

| Raw dataset parameters for driven pile bearing capacity prediction | | | | | | | | | | |
| --- | --- | --- | --- | --- | --- | --- | --- | --- | --- | --- |
| X1 | X2 | X3 | X4 | X5 | X6 | X7 | X8 | X9 | X10 | Output |
| 0.3 | 8.65 | 3.4 | 5.25 | 0 | 3.4 | 3.47 | 3.42 | 8.65 | 6.75 | 559.8 |
| 0.4 | 13.15 | 4.35 | 8 | 0.8 | 2.05 | 3.45 | 2.25 | 13.15 | 7.52 | 1392 |
| 0.3 | 8.75 | 3.4 | 5.35 | 0 | 3.4 | 3.57 | 3.42 | 8.75 | 6.78 | 661.6 |
| 0.3 | 8.75 | 3.4 | 5.35 | 0 | 3.4 | 3.57 | 3.42 | 8.75 | 6.78 | 661.6 |
| 0.3 | 8.6 | 3.4 | 5.2 | 0 | 3.4 | 3.45 | 3.45 | 8.6 | 6.73 | 610.7 |
| 0.4 | 15.41 | 5.72 | 8 | 1.69 | 0.68 | 4.12 | 1.03 | 15.41 | 7.5 | 1344 |
| 0.3 | 8.65 | 3.4 | 5.25 | 0 | 3.4 | 3.49 | 3.44 | 8.65 | 6.75 | 555.3 |
| 0.3 | 8.62 | 3.4 | 5.22 | 0 | 3.4 | 3.44 | 3.42 | 8.62 | 6.738979118 | 559.8 |
| 0.4 | 13.61 | 4.45 | 8 | 1.16 | 1.95 | 3.55 | 1.99 | 13.61 | 7.680014695 | 1224.8 |
| 0.4 | 11.45 | 3.85 | 7.6 | 0 | 2.95 | 3.67 | 3.27 | 11.45 | 7.15 | 1425 |
| 0.4 | 13.25 | 4.35 | 8 | 0.9 | 2.05 | 3.37 | 2.07 | 13.25 | 7.58 | 1395 |
| 0.4 | 13.37 | 4.35 | 8 | 1.02 | 2.05 | 3.47 | 2.05 | 13.37 | 7.6421092 | 1395 |
| 0.4 | 13.38 | 4.35 | 8 | 1.03 | 2.05 | 3.48 | 2.05 | 13.38 | 7.647608371 | 1395 |
| 0.3 | 8.6 | 3.4 | 5.2 | 0 | 3.4 | 3.4 | 3.4 | 8.6 | 6.73 | 559 |
| 0.4 | 5.76 | 4.1 | 1.66 | 0 | 2.7 | 3.21 | 2.75 | 5.76 | 4.52 | 423.9 |
| 0.3 | 8.6 | 3.4 | 5.2 | 0 | 3.4 | 3.43 | 3.43 | 8.6 | 6.73 | 610.7 |
| 0.4 | 13.39 | 4.45 | 8 | 0.94 | 1.95 | 3.34 | 2 | 13.39 | 7.559746079 | 1128.6 |
| 0.3 | 8.64 | 3.4 | 5.24 | 0 | 3.4 | 3.46 | 3.42 | 8.64 | 6.75 | 559.8 |
| 0.4 | 13.42 | 4.35 | 8 | 1.07 | 2.05 | 3.52 | 2.05 | 13.42 | 7.6695231 | 1082.3 |
| 0.4 | 6.18 | 4.1 | 2.08 | 0 | 2.7 | 3.63 | 2.75 | 6.18 | 4.86 | 432 |
| 0.3 | 8.62 | 3.4 | 5.22 | 0 | 3.4 | 3.46 | 3.44 | 8.62 | 6.74 | 661.6 |
| 0.4 | 12.95 | 4.35 | 8 | 0.6 | 2.05 | 3.5 | 2.5 | 12.95 | 7.403474903 | 1297.8 |
| 0.3 | 8.7 | 3.4 | 5.3 | 0 | 3.4 | 3.5 | 3.4 | 8.7 | 6.76 | 559.8 |
| 0.3 | 8.6 | 3.4 | 5.2 | 0 | 3.4 | 3.38 | 3.38 | 8.6 | 6.73255814 | 610.7 |
| 0.4 | 13.25 | 4.35 | 8 | 0.9 | 2.05 | 3.43 | 2.13 | 13.25 | 7.575471698 | 1395 |
| 0.4 | 13.43 | 4.35 | 8 | 1.08 | 2.05 | 3.53 | 2.05 | 13.43 | 7.67 | 1248 |
| 0.4 | 10.7 | 3.4 | 7.3 | 0 | 3.4 | 3.51 | 3.41 | 10.7 | 7.28 | 1068.8 |
| 0.4 | 11.72 | 3.45 | 8 | 0.27 | 2.95 | 3.63 | 2.96 | 11.72 | 7.57 | 1152 |
| 0.4 | 12.65 | 4.35 | 8 | 0.3 | 2.05 | 3.5 | 2.8 | 12.65 | 7.223320158 | 1297.8 |
| 0.4 | 13.41 | 4.35 | 8 | 1.06 | 2.05 | 3.56 | 2.1 | 13.41 | 7.66 | 1224.8 |
| 0.4 | 13.44 | 4.45 | 8 | 0.99 | 1.95 | 3.38 | 1.99 | 13.44 | 7.59 | 1128.6 |
| 0.4 | 13.48 | 4.35 | 8 | 1.13 | 2.05 | 3.63 | 2.1 | 13.48 | 7.702151335 | 1224.8 |
| 0.3 | 8.6 | 3.4 | 5.2 | 0 | 3.4 | 3.43 | 3.43 | 8.6 | 6.73 | 559.8 |
| 0.4 | 13.4 | 4.35 | 8 | 1.05 | 2.05 | 3.51 | 2.06 | 13.4 | 7.66 | 1248 |
| 0.4 | 12.53 | 4.35 | 8 | 0.18 | 2.05 | 3.38 | 2.8 | 12.53 | 7.15 | 1473 |
| 0.3 | 8.7 | 3.4 | 5.3 | 0 | 3.4 | 3.51 | 3.41 | 8.7 | 6.76 | 661.6 |
| 0.4 | 13.4 | 4.35 | 8 | 1.05 | 2.05 | 3.5 | 2.05 | 13.4 | 7.66 | 1152 |
| 0.4 | 13.35 | 4.35 | 8 | 1 | 2.05 | 3.48 | 2.08 | 13.35 | 7.63 | 1395 |
| 0.4 | 10.72 | 3.4 | 7.32 | 0 | 3.4 | 3.57 | 3.45 | 10.72 | 7.28 | 1032.4 |
| 0.4 | 11.61 | 3.45 | 8 | 0.16 | 2.95 | 3.53 | 2.97 | 11.61 | 7.495693368 | 1056 |
| 0.3 | 8.6 | 3.4 | 5.2 | 0 | 3.4 | 3.42 | 3.42 | 8.6 | 6.73 | 559.8 |
| 0.3 | 8.6 | 3.4 | 5.2 | 0 | 3.4 | 3.42 | 3.42 | 8.6 | 6.73 | 661.6 |
| 0.4 | 13.37 | 4.35 | 8 | 1.02 | 2.05 | 3.47 | 4.05 | 13.37 | 7.64 | 1318 |
| 0.4 | 11.75 | 3.45 | 8 | 0.3 | 2.95 | 3.65 | 2.95 | 11.75 | 7.59 | 1152 |
| 0.3 | 8.6 | 3.4 | 5.2 | 0 | 3.4 | 3.43 | 3.43 | 8.6 | 6.73 | 585.35 |
| 0.4 | 12.45 | 4.25 | 8 | 0.2 | 2.15 | 3.55 | 2.95 | 12.45 | 7.2 | 1392 |
| 0.3 | 8.65 | 3.4 | 5.25 | 0 | 3.4 | 3.49 | 3.44 | 8.65 | 6.75 | 610.7 |
| 0.4 | 13.31 | 4.35 | 8 | 0.96 | 2.05 | 3.42 | 2.06 | 13.31 | 7.608940646 | 1119.7 |
| 0.4 | 11.65 | 3.45 | 8 | 0.2 | 2.95 | 3.58 | 2.98 | 11.65 | 7.52 | 1068.8 |
| 0.4 | 12.71 | 4.05 | 8 | 0.66 | 2.35 | 3.46 | 2.4 | 12.71 | 7.56 | 1318 |
| 0.3 | 8.6 | 3.4 | 5.2 | 0 | 3.4 | 3.41 | 3.41 | 8.6 | 6.73 | 610.7 |
| 0.3 | 8.72 | 3.4 | 5.32 | 0 | 3.4 | 3.55 | 3.43 | 8.72 | 6.77 | 661.6 |
| 0.3 | 8.55 | 3.4 | 5.15 | 0 | 3.4 | 3.36 | 3.41 | 8.55 | 6.72 | 610.7 |
| 0.4 | 12.15 | 4.75 | 7.4 | 0 | 2.05 | 3.55 | 3.35 | 12.15 | 6.76 | 1440 |
| 0.4 | 10.75 | 3.4 | 7.35 | 0 | 3.4 | 3.57 | 3.42 | 10.75 | 7.29 | 1119.7 |
| 0.3 | 8.6 | 3.4 | 5.2 | 0 | 3.4 | 3.42 | 3.42 | 8.6 | 6.73 | 610.7 |
| 0.4 | 13.48 | 4.45 | 8 | 1.03 | 1.95 | 3.43 | 2 | 13.48 | 7.61 | 1128.6 |
| 0.4 | 6.1 | 4.1 | 2 | 0 | 2.7 | 3.56 | 2.76 | 6.1 | 4.8 | 620 |
| 0.4 | 11.66 | 3.5 | 8 | 0.16 | 2.9 | 3.47 | 2.91 | 11.66 | 7.47 | 1056 |
| 0.4 | 10.71 | 3.4 | 7.31 | 0 | 3.4 | 3.56 | 3.45 | 10.71 | 7.28 | 1224.8 |
| 0.4 | 13.1 | 4.35 | 8 | 0.75 | 2.05 | 3.45 | 2.3 | 13.1 | 7.490458015 | 1323.2 |
| 0.4 | 13.36 | 4.35 | 8 | 1.01 | 2.05 | 3.46 | 2.05 | 13.36 | 7.64 | 1473 |
| 0.4 | 13.31 | 4.35 | 8 | 0.96 | 2.05 | 3.42 | 2.06 | 13.31 | 7.608940646 | 1244 |
| 0.4 | 13.4 | 4.35 | 8 | 1.05 | 2.05 | 3.55 | 2.1 | 13.4 | 7.66 | 1473 |
| 0.4 | 13 | 4.2 | 8 | 0.8 | 2.2 | 3.49 | 2.29 | 13 | 7.58 | 1395 |
| 0.3 | 8.65 | 3.4 | 5.25 | 0 | 3.4 | 3.46 | 3.41 | 8.65 | 6.75 | 610.7 |
| 0.4 | 13.45 | 4.45 | 8 | 1 | 1.95 | 3.4 | 2 | 13.45 | 7.59 | 1128.6 |
| 0.4 | 10.71 | 3.4 | 7.31 | 0 | 3.4 | 3.51 | 3.4 | 10.71 | 7.28 | 1395 |
| 0.4 | 12.65 | 4.05 | 8 | 0.6 | 2.35 | 3.41 | 2.41 | 12.65 | 7.52 | 1240 |
| 0.3 | 8.65 | 3.4 | 5.25 | 0 | 3.4 | 3.48 | 3.43 | 8.65 | 6.75 | 559.8 |
| 0.4 | 13.39 | 4.35 | 8 | 1.04 | 2.05 | 3.49 | 2.05 | 13.39 | 7.65 | 1395 |
| 0.4 | 11.52 | 3.45 | 8 | 0.07 | 2.95 | 3.42 | 2.95 | 11.52 | 7.44 | 1240 |
| 0.4 | 13.35 | 4.35 | 8 | 1 | 2.05 | 3.46 | 2.06 | 13.35 | 7.63 | 1344 |
| 0.3 | 8.7 | 3.4 | 5.3 | 0 | 3.4 | 3.52 | 3.42 | 8.7 | 6.764367816 | 661.6 |
| 0.3 | 8.64 | 3.4 | 5.24 | 0 | 3.4 | 3.48 | 3.44 | 8.64 | 6.75 | 610.7 |
| 0.3 | 8.7 | 3.4 | 5.3 | 0 | 3.4 | 3.5 | 3.4 | 8.7 | 6.76 | 611.7 |
| 0.3 | 8.61 | 3.4 | 5.21 | 0 | 3.4 | 3.44 | 3.43 | 8.61 | 6.74 | 508.5 |
| 0.4 | 6 | 4.1 | 1.9 | 0 | 2.7 | 3.43 | 2.73 | 6 | 4.716666667 | 620 |
| 0.4 | 5.82 | 4.1 | 1.72 | 0 | 2.7 | 3.27 | 2.75 | 5.82 | 4.57 | 423.9 |
| 0.4 | 12.35 | 4.75 | 7.6 | 0 | 2.05 | 3.49 | 3.09 | 12.35 | 6.807692308 | 1473 |
| 0.4 | 13.4 | 4.35 | 8 | 1.05 | 2.05 | 3.55 | 2.1 | 13.4 | 7.66 | 1221.5 |
| 0.4 | 11.54 | 3.45 | 8 | 0.09 | 2.95 | 3.44 | 2.95 | 11.54 | 7.45017331 | 1318 |
| 0.3 | 8.62 | 3.4 | 5.22 | 0 | 3.4 | 3.44 | 3.42 | 8.62 | 6.74 | 610.7 |
| 0.4 | 11.7 | 3.5 | 8 | 0.2 | 2.9 | 3.5 | 2.9 | 11.7 | 7.5 | 1056 |
| 0.4 | 13.25 | 4.35 | 8 | 0.9 | 2.05 | 3.38 | 2.08 | 13.25 | 7.58 | 1395 |
| 0.4 | 13.39 | 4.35 | 8 | 1.04 | 2.05 | 3.54 | 2.1 | 13.39 | 7.65 | 1224.8 |
| 0.4 | 13.37 | 4.35 | 8 | 1.02 | 2.05 | 3.47 | 2.05 | 13.37 | 7.64 | 1152 |
| 0.3 | 8.65 | 3.4 | 5.25 | 0 | 3.4 | 3.49 | 3.44 | 8.65 | 6.75 | 559.8 |
| 0.3 | 8.65 | 3.4 | 5.25 | 0 | 3.4 | 3.51 | 3.46 | 8.65 | 6.75 | 559.8 |
| 0.4 | 13.25 | 4.35 | 8 | 0.9 | 2.05 | 3.4 | 2.1 | 13.25 | 7.58 | 1395 |
| 0.4 | 13.21 | 4.25 | 8 | 0.96 | 2.15 | 3.53 | 2.17 | 13.21 | 7.65 | 1344 |
| 0.3 | 8.62 | 3.4 | 5.22 | 0 | 3.4 | 3.46 | 3.44 | 8.62 | 6.738979118 | 661.6 |
| 0.4 | 11.65 | 3.45 | 8 | 0.2 | 2.95 | 3.56 | 2.96 | 11.65 | 7.52 | 1152 |
| 0.4 | 12.95 | 4.35 | 8 | 0.6 | 2.05 | 3.46 | 2.46 | 12.95 | 7.403474903 | 1395 |
| 0.3 | 8.58 | 3.4 | 5.18 | 0 | 3.4 | 3.36 | 3.38 | 8.58 | 6.73 | 559.8 |
| 0.4 | 13.43 | 4.35 | 8 | 1.08 | 2.05 | 3.56 | 2.08 | 13.43 | 7.67 | 1224.8 |
| 0.3 | 8.65 | 3.4 | 5.25 | 0 | 3.4 | 3.47 | 3.42 | 8.65 | 6.75 | 610.7 |
| 0.4 | 11.7 | 3.45 | 8 | 0.25 | 2.95 | 3.6 | 2.95 | 11.7 | 7.553418803 | 960 |
| 0.4 | 13.4 | 4.35 | 8 | 1.05 | 2.05 | 3.52 | 2.07 | 13.4 | 7.66 | 1248 |
| 0.4 | 12.35 | 4.25 | 8 | 0.1 | 2.15 | 3.54 | 3.04 | 12.35 | 7.14 | 1551 |
| 0.4 | 13.45 | 4.35 | 8 | 1.1 | 2.05 | 3.55 | 2.05 | 13.45 | 7.69 | 1425 |
| 0.4 | 13.53 | 4.35 | 8 | 1.18 | 2.05 | 3.66 | 2.08 | 13.53 | 7.73 | 1056 |
| 0.3 | 8.65 | 3.4 | 5.25 | 0 | 3.4 | 3.47 | 3.42 | 8.65 | 6.75 | 610.7 |
| 0.4 | 13.29 | 4.35 | 8 | 0.94 | 2.05 | 3.49 | 2.15 | 13.29 | 7.597817908 | 1395 |
| 0.4 | 13.27 | 4.25 | 8 | 1.02 | 2.15 | 3.58 | 2.16 | 13.27 | 7.68 | 1248 |
| 0.4 | 6.3 | 4.1 | 2.2 | 0 | 2.7 | 3.72 | 2.72 | 6.3 | 4.944444444 | 610.7 |
| 0.4 | 13.33 | 4.35 | 8 | 0.98 | 2.05 | 3.44 | 2.06 | 13.33 | 7.620030008 | 1344 |
| 0.4 | 11.35 | 3.85 | 7.5 | 0 | 2.95 | 3.66 | 3.36 | 11.35 | 7.13 | 1425 |
| 0.3 | 8.6 | 3.4 | 5.2 | 0 | 3.4 | 3.48 | 3.48 | 8.6 | 6.73 | 611.6 |
| 0.4 | 12.35 | 4.75 | 7.6 | 0 | 2.05 | 3.49 | 3.09 | 12.35 | 6.807692308 | 1473 |
| 0.4 | 11.55 | 3.45 | 8 | 0.1 | 2.95 | 3.54 | 3.04 | 11.55 | 7.46 | 1017.9 |
| 0.3 | 8.6 | 3.4 | 5.2 | 0 | 3.4 | 3.43 | 3.43 | 8.6 | 6.73 | 610.7 |
| 0.3 | 8.62 | 3.4 | 5.22 | 0 | 3.4 | 3.44 | 3.42 | 8.62 | 6.74 | 617 |
| 0.4 | 13.32 | 4.35 | 8 | 0.97 | 2.05 | 3.47 | 2.1 | 13.32 | 7.61 | 1395 |
| 0.4 | 13.41 | 4.35 | 8 | 1.06 | 2.05 | 3.52 | 2.06 | 13.41 | 7.66 | 1344 |
| 0.3 | 8.7 | 3.4 | 5.3 | 0 | 3.4 | 3.5 | 3.4 | 8.7 | 6.76 | 559.8 |
| 0.4 | 5.94 | 4.1 | 1.84 | 0 | 2.7 | 3.39 | 2.75 | 5.94 | 4.67 | 423.9 |
| 0.4 | 12.85 | 4.05 | 8 | 0.8 | 2.35 | 3.56 | 2.36 | 12.85 | 7.64 | 1318 |
| 0.3 | 8.7 | 3.4 | 5.3 | 0 | 3.4 | 3.5 | 3.4 | 8.7 | 6.76 | 661.6 |
| 0.4 | 13.25 | 4.25 | 8 | 1 | 2.15 | 3.56 | 2.16 | 13.25 | 7.669811321 | 1248 |
| 0.4 | 13.3 | 4.35 | 8 | 0.95 | 2.05 | 3.42 | 2.07 | 13.3 | 7.6 | 1119.7 |
| 0.4 | 12.65 | 4.25 | 8 | 0.4 | 2.15 | 3.59 | 2.79 | 12.65 | 7.32 | 1551 |
| 0.3 | 8.7 | 3.4 | 5.3 | 0 | 3.4 | 3.52 | 3.42 | 8.7 | 6.76 | 508.9 |
| 0.4 | 6.02 | 4.1 | 1.92 | 0 | 2.7 | 3.44 | 2.72 | 6.02 | 4.73 | 712.5 |
| 0.4 | 13.34 | 4.35 | 8 | 0.99 | 2.05 | 3.44 | 2.05 | 13.34 | 7.63 | 1395 |
| 0.3 | 8.6 | 3.4 | 5.2 | 0 | 3.4 | 3.43 | 3.43 | 8.6 | 6.73 | 610.7 |
| 0.4 | 10.68 | 3.4 | 7.28 | 0 | 3.4 | 3.53 | 3.45 | 10.68 | 7.27 | 1032.4 |
| 0.4 | 5.85 | 4.1 | 1.75 | 0 | 2.7 | 3.26 | 2.71 | 5.85 | 4.59 | 508.9 |
| 0.4 | 13.25 | 4.35 | 8 | 0.9 | 2.05 | 3.42 | 2.12 | 13.25 | 7.575471698 | 1395 |
| 0.3 | 8.6 | 3.4 | 5.2 | 0 | 3.4 | 3.42 | 3.42 | 8.6 | 6.73 | 610.7 |
| 0.3 | 8.65 | 3.4 | 5.25 | 0 | 3.4 | 3.47 | 3.42 | 8.65 | 6.75 | 559.8 |
| 0.4 | 10.71 | 3.4 | 7.31 | 0 | 3.4 | 3.54 | 3.43 | 10.71 | 7.28 | 1032.8 |
| 0.3 | 8.65 | 3.4 | 5.25 | 0 | 3.4 | 3.48 | 3.43 | 8.65 | 6.748554913 | 508.9 |
| 0.4 | 13.33 | 4.35 | 8 | 0.98 | 2.05 | 3.45 | 2.07 | 13.33 | 7.620030008 | 1395 |
| 0.4 | 13.42 | 4.35 | 8 | 1.07 | 2.05 | 3.55 | 2.08 | 13.42 | 7.6695231 | 1224.8 |
| 0.4 | 12.75 | 4.05 | 8 | 0.7 | 2.35 | 3.48 | 2.38 | 12.75 | 7.58 | 1240 |
| 0.4 | 13.36 | 4.35 | 8 | 1.01 | 2.05 | 3.51 | 2.1 | 13.36 | 7.64 | 1473 |
| 0.4 | 11.75 | 3.45 | 8 | 0.3 | 2.95 | 3.65 | 2.95 | 11.75 | 7.59 | 1017.9 |
| 0.4 | 13.55 | 4.45 | 8 | 1.1 | 1.95 | 3.5 | 2 | 13.55 | 7.65 | 1128.6 |
| 0.3 | 8.65 | 3.4 | 5.25 | 0 | 3.4 | 3.49 | 3.44 | 8.65 | 6.75 | 610.7 |
| 0.4 | 5.9 | 4.1 | 1.8 | 0 | 2.7 | 3.39 | 2.79 | 5.9 | 4.64 | 620 |
| 0.3 | 8.65 | 3.4 | 5.25 | 0 | 3.4 | 3.46 | 3.41 | 8.65 | 6.75 | 407.2 |
| 0.3 | 8.65 | 3.4 | 5.25 | 0 | 3.4 | 3.48 | 3.43 | 8.65 | 6.75 | 610.7 |
| 0.4 | 11.63 | 3.45 | 8 | 0.18 | 2.95 | 3.55 | 2.97 | 11.63 | 7.51 | 1344 |
| 0.3 | 8.62 | 3.4 | 5.22 | 0 | 3.4 | 3.44 | 3.42 | 8.62 | 6.74 | 661.6 |
| 0.4 | 13.36 | 4.35 | 8 | 1.01 | 2.05 | 3.46 | 2.05 | 13.36 | 7.64 | 1473 |
| 0.4 | 12.35 | 4.75 | 7.6 | 0 | 2.05 | 3.46 | 3.06 | 12.35 | 6.807692308 | 1473 |
| 0.3 | 8.65 | 3.4 | 5.25 | 0 | 3.4 | 3.46 | 3.41 | 8.65 | 6.748554913 | 532.4 |
| 0.4 | 11.7 | 5.4 | 6.3 | 0 | 2.15 | 3.52 | 1.06 | 14.7 | 5.5 | 1056 |
| 0.4 | 10.7 | 3.4 | 7.3 | 0 | 3.4 | 3.5 | 3.4 | 10.7 | 7.28 | 960 |
| 0.4 | 13.45 | 4.35 | 8 | 1.1 | 2.05 | 3.54 | 2.04 | 13.45 | 7.685873606 | 1323.2 |
| 0.4 | 13.25 | 4.25 | 8 | 1 | 2.15 | 3.55 | 2.15 | 13.25 | 7.669811321 | 1248 |
| 0.4 | 11.66 | 4.45 | 7.21 | 0 | 2.35 | 3.41 | 2.4 | 11.66 | 6.83 | 1318 |
| 0.4 | 10.7 | 3.4 | 7.3 | 0 | 3.4 | 3.5 | 3.4 | 10.7 | 7.28 | 1152 |
| 0.4 | 11.55 | 3.5 | 8 | 0.05 | 2.9 | 3.36 | 2.91 | 11.55 | 7.4 | 1056 |
| 0.4 | 12.65 | 4.35 | 8 | 0.3 | 2.05 | 3.45 | 2.75 | 12.65 | 7.22 | 1473 |
| 0.4 | 12.2 | 4.75 | 7.45 | 0 | 2.05 | 3.67 | 3.42 | 12.2 | 6.774590164 | 1440 |
| 0.4 | 12.35 | 4.75 | 7.6 | 0 | 2.05 | 3.65 | 3.25 | 12.35 | 6.81 | 1440 |
| 0.4 | 12.65 | 4.25 | 8 | 0.4 | 2.15 | 3.55 | 2.75 | 12.65 | 7.32 | 1392 |
| 0.4 | 6.05 | 4.1 | 1.95 | 0 | 2.7 | 3.49 | 2.74 | 6.05 | 4.76 | 712.5 |
| 0.4 | 5.81 | 4.1 | 1.71 | 0 | 2.7 | 3.26 | 2.75 | 5.81 | 4.56 | 423.9 |
| 0.4 | 13.45 | 4.35 | 8 | 1.1 | 2.05 | 3.6 | 2.1 | 13.45 | 7.69 | 1224.8 |
| 0.3 | 8.65 | 3.4 | 5.25 | 0 | 3.4 | 3.48 | 3.43 | 8.65 | 6.75 | 661.6 |
| 0.4 | 13.37 | 4.35 | 8 | 1.02 | 2.05 | 3.48 | 2.06 | 13.37 | 7.6421092 | 1248 |
| 0.4 | 11.7 | 3.5 | 8 | 0.2 | 2.9 | 3.5 | 2.9 | 11.7 | 7.5 | 1056 |
| 0.4 | 10.68 | 3.4 | 7.28 | 0 | 3.4 | 3.53 | 3.45 | 10.68 | 7.27 | 1032.4 |
| 0.4 | 11.62 | 3.45 | 8 | 0.17 | 2.95 | 3.54 | 2.97 | 11.62 | 7.5 | 1056 |
| 0.3 | 8.6 | 3.4 | 5.2 | 0 | 3.4 | 3.43 | 3.43 | 8.6 | 6.73 | 661.6 |
| 0.3 | 8.62 | 3.4 | 5.22 | 0 | 3.4 | 3.44 | 3.42 | 8.62 | 6.738979118 | 617 |
| 0.3 | 8.65 | 3.4 | 5.25 | 0 | 3.4 | 3.46 | 3.41 | 8.65 | 6.75 | 532.4 |
| 0.4 | 13.25 | 4.25 | 8 | 1 | 2.15 | 3.56 | 2.16 | 13.25 | 7.67 | 1344 |
| 0.3 | 8.6 | 3.4 | 5.2 | 0 | 3.4 | 3.4 | 3.4 | 8.6 | 6.73 | 559.8 |
| 0.4 | 13.4 | 4.35 | 8 | 1.05 | 2.05 | 3.55 | 2.1 | 13.4 | 7.66 | 1224.8 |
| 0.4 | 11.6 | 3.45 | 8 | 0.15 | 2.95 | 3.5 | 2.95 | 11.6 | 7.49 | 1240 |
| 0.4 | 10.7 | 3.4 | 7.3 | 0 | 3.4 | 3.54 | 3.44 | 10.7 | 7.28 | 967 |
| 0.4 | 10.75 | 3.4 | 7.35 | 0 | 3.4 | 3.56 | 3.41 | 10.75 | 7.29 | 1052.4 |
| 0.3 | 8.6 | 3.4 | 5.2 | 0 | 3.4 | 3.43 | 3.43 | 8.6 | 6.73 | 600.7 |
| 0.4 | 13.43 | 4.35 | 8 | 1.08 | 2.05 | 3.54 | 2.06 | 13.43 | 7.674981385 | 1395 |
| 0.4 | 13.34 | 4.35 | 8 | 0.99 | 2.05 | 3.49 | 2.1 | 13.34 | 7.63 | 1224.8 |
| 0.4 | 12.15 | 4.75 | 7.4 | 0 | 2.05 | 3.52 | 3.32 | 12.15 | 6.76 | 1425 |
| 0.3 | 8.67 | 3.4 | 5.27 | 0 | 3.4 | 3.5 | 3.43 | 8.67 | 6.75 | 610.7 |
| 0.4 | 10.62 | 3.4 | 7.22 | 0 | 3.4 | 3.47 | 3.45 | 10.62 | 7.26 | 1128.6 |
| 0.4 | 6.1 | 4.1 | 2 | 0 | 2.7 | 3.55 | 2.75 | 6.1 | 4.8 | 610.7 |
| 0.4 | 13.45 | 4.35 | 8 | 1.1 | 2.05 | 3.6 | 2.1 | 13.45 | 7.69 | 1395 |
| 0.4 | 11.21 | 3.85 | 7.36 | 0 | 2.95 | 3.66 | 3.5 | 11.21 | 7.1 | 1344 |
| 0.4 | 13.37 | 4.35 | 8 | 1.02 | 2.05 | 3.47 | 2.05 | 13.37 | 7.64 | 1473 |
| 0.4 | 11.64 | 3.45 | 8 | 0.19 | 2.95 | 3.56 | 2.97 | 11.64 | 7.52 | 1318 |
| 0.3 | 8.6 | 3.4 | 5.2 | 0 | 3.4 | 3.42 | 3.42 | 8.6 | 6.73 | 617 |
| 0.3 | 8.62 | 3.4 | 5.22 | 0 | 3.4 | 3.44 | 3.42 | 8.62 | 6.74 | 610.7 |
| 0.4 | 13.41 | 4.35 | 8 | 1.06 | 2.05 | 3.56 | 2.1 | 13.41 | 7.66 | 1224.8 |
| 0.4 | 13.3 | 4.35 | 8 | 0.95 | 2.05 | 3.41 | 2.06 | 13.3 | 7.6 | 1110.6 |
| 0.4 | 11.57 | 3.45 | 8 | 0.12 | 2.95 | 3.47 | 2.95 | 11.57 | 7.47 | 1318 |
| 0.4 | 11.7 | 3.45 | 8 | 0.25 | 2.95 | 3.62 | 2.97 | 11.7 | 7.55 | 1170.6 |
| 0.3 | 8.6 | 3.4 | 5.2 | 0 | 3.4 | 3.4 | 3.4 | 8.6 | 6.73 | 559.8 |
| 0.4 | 10.68 | 3.4 | 7.28 | 0 | 3.4 | 3.48 | 3.4 | 10.68 | 7.27 | 1318 |
| 0.4 | 13.4 | 4.35 | 8 | 1.05 | 2.05 | 3.53 | 2.08 | 13.4 | 7.65858209 | 1473 |
| 0.4 | 10.8 | 3.4 | 7.4 | 0 | 3.4 | 3.61 | 3.41 | 10.8 | 7.296296296 | 1088.8 |
| 0.4 | 12.05 | 4.65 | 7.4 | 0 | 2.15 | 3.59 | 3.39 | 12.05 | 6.8 | 1551 |
| 0.3 | 8.66 | 3.4 | 5.26 | 0 | 3.4 | 3.49 | 3.43 | 8.66 | 6.75 | 508.9 |
| 0.3 | 8.65 | 3.4 | 5.25 | 0 | 3.4 | 3.46 | 3.41 | 8.65 | 6.75 | 559.8 |
| 0.4 | 13.25 | 4.35 | 8 | 0.9 | 2.05 | 3.4 | 2.1 | 13.25 | 7.575471698 | 1473 |
| 0.4 | 11.67 | 3.45 | 8 | 0.22 | 2.95 | 3.57 | 2.95 | 11.67 | 7.53 | 1318 |
| 0.3 | 8.65 | 3.4 | 5.25 | 0 | 3.4 | 3.47 | 3.42 | 8.65 | 6.75 | 610.7 |
| 0.4 | 13.33 | 4.35 | 8 | 0.98 | 2.05 | 3.48 | 2.1 | 13.33 | 7.62 | 1224.8 |
| 0.4 | 13.35 | 4.35 | 8 | 1 | 2.05 | 3.47 | 2.07 | 13.35 | 7.63 | 1344 |
| 0.3 | 8.6 | 3.4 | 5.2 | 0 | 3.4 | 3.42 | 3.42 | 8.6 | 6.73 | 660.6 |
| 0.4 | 13.57 | 4.35 | 8 | 1.22 | 2.05 | 3.67 | 2.05 | 13.57 | 7.75 | 1248 |
| 0.4 | 13.38 | 4.35 | 8 | 1.03 | 2.05 | 3.53 | 2.1 | 13.38 | 7.65 | 1224.8 |
| 0.4 | 11.15 | 3.85 | 7.3 | 0 | 2.95 | 3.7 | 3.6 | 11.15 | 7.08 | 1440 |
| 0.4 | 13.46 | 4.35 | 8 | 1.11 | 2.05 | 3.56 | 2.05 | 13.46 | 7.69 | 1128.6 |
| 0.4 | 5.6 | 4.1 | 1.5 | 0 | 2.7 | 3.04 | 2.74 | 5.6 | 4.38 | 508.9 |
| 0.4 | 11.52 | 3.45 | 8 | 0.07 | 2.95 | 3.42 | 2.95 | 11.52 | 7.44 | 1240 |
| 0.3 | 8.63 | 3.4 | 5.23 | 0 | 3.4 | 3.44 | 3.41 | 8.63 | 6.742178447 | 610.7 |
| 0.4 | 13.31 | 4.35 | 8 | 0.96 | 2.05 | 3.46 | 2.1 | 13.31 | 7.61 | 1395 |
| 0.3 | 8.62 | 3.4 | 5.22 | 0 | 3.4 | 3.44 | 3.42 | 8.62 | 6.74 | 610.7 |
| 0.3 | 8.66 | 3.4 | 5.26 | 0 | 3.4 | 3.47 | 3.41 | 8.66 | 6.75 | 559.8 |
| 0.4 | 12.45 | 4.25 | 8 | 0.2 | 2.15 | 3.56 | 2.96 | 12.45 | 7.2 | 1392 |
| 0.3 | 8.65 | 3.4 | 5.25 | 0 | 3.4 | 3.48 | 3.43 | 8.65 | 6.75 | 559.8 |
| 0.4 | 13.46 | 4.35 | 8 | 1.11 | 2.05 | 3.6 | 2.09 | 13.46 | 7.691307578 | 1128.6 |
| 0.3 | 8.7 | 3.4 | 5.3 | 0 | 3.4 | 3.53 | 3.43 | 8.7 | 6.76 | 559.8 |
| 0.3 | 8.6 | 3.4 | 5.2 | 0 | 3.4 | 3.45 | 3.45 | 8.6 | 6.73 | 559.8 |
| 0.4 | 11.67 | 3.45 | 8 | 0.22 | 2.95 | 3.59 | 2.97 | 11.67 | 7.53 | 1152 |
| 0.4 | 13.38 | 4.35 | 8 | 1.03 | 2.05 | 3.48 | 2.05 | 13.38 | 7.65 | 1344 |
| 0.4 | 13.39 | 4.45 | 8 | 0.94 | 1.95 | 3.49 | 2.15 | 13.39 | 7.56 | 1318 |
| 0.4 | 8.91 | 3.55 | 5.36 | 0 | 3.25 | 3.41 | 3.25 | 8.91 | 6.71 | 930 |
| 0.3 | 8.65 | 3.4 | 5.25 | 0 | 3.4 | 3.47 | 3.42 | 8.65 | 6.75 | 585.4 |
| 0.3 | 8.63 | 3.4 | 5.23 | 0 | 3.4 | 3.47 | 3.44 | 8.63 | 6.74 | 559.8 |
| 0.3 | 8.6 | 3.4 | 5.2 | 0 | 3.4 | 3.45 | 3.45 | 8.6 | 6.73 | 610.7 |
| 0.3 | 8.65 | 3.4 | 5.25 | 0 | 3.4 | 3.47 | 3.42 | 8.65 | 6.75 | 559.8 |
| 0.4 | 12.06 | 4.75 | 7.31 | 0 | 2.05 | 3.61 | 3.5 | 12.06 | 6.74 | 1440 |
| 0.4 | 13.43 | 4.35 | 8 | 1.08 | 2.05 | 3.56 | 2.08 | 13.43 | 7.674981385 | 1344 |
| 0.4 | 6.2 | 4.1 | 2.1 | 0 | 2.7 | 3.63 | 2.73 | 6.2 | 4.87 | 480 |
| 0.4 | 10.68 | 3.4 | 7.28 | 0 | 3.4 | 3.48 | 3.4 | 10.68 | 7.27 | 1318 |
| 0.4 | 13.35 | 4.35 | 8 | 1 | 2.05 | 3.52 | 2.12 | 13.35 | 7.63 | 1395 |
| 0.4 | 12 | 4.65 | 7.35 | 0 | 2.15 | 3.55 | 3.4 | 12 | 6.79 | 1392 |
| 0.4 | 8.69 | 3.45 | 5.24 | 0 | 3.35 | 3.44 | 3.4 | 8.69 | 6.72 | 1240 |
| 0.4 | 10.73 | 3.4 | 7.33 | 0 | 3.4 | 3.55 | 3.42 | 10.73 | 7.281919851 | 1094.25 |
| 0.4 | 13.4 | 4.35 | 8 | 1.05 | 2.05 | 3.5 | 2.05 | 13.4 | 7.66 | 1344 |
| 0.3 | 8.63 | 3.4 | 5.23 | 0 | 3.4 | 3.44 | 3.41 | 8.63 | 6.74 | 585.35 |
| 0.4 | 13.05 | 4.35 | 8 | 0.7 | 2.05 | 3.49 | 2.39 | 13.05 | 7.46 | 1392 |
| 0.4 | 13.37 | 4.35 | 8 | 1.02 | 2.05 | 3.48 | 2.06 | 13.37 | 7.6421092 | 1248 |
| 0.4 | 13.4 | 4.35 | 8 | 1.05 | 2.05 | 3.5 | 2.05 | 13.4 | 7.66 | 1128.6 |
| 0.4 | 13.45 | 4.35 | 8 | 1.1 | 2.05 | 3.55 | 2.05 | 13.45 | 7.69 | 1119.7 |
| 0.4 | 13.16 | 4.25 | 8 | 0.91 | 2.15 | 3.56 | 2.25 | 13.16 | 7.62 | 1473 |
| 0.4 | 12 | 4.75 | 7.25 | 0 | 2.05 | 3.62 | 3.57 | 12 | 6.73 | 1425 |
| 0.3 | 8.65 | 3.4 | 5.25 | 0 | 3.4 | 3.47 | 3.42 | 8.65 | 6.75 | 610.7 |
| 0.4 | 11.58 | 3.45 | 8 | 0.13 | 2.95 | 3.51 | 2.98 | 11.58 | 7.48 | 1344 |
| 0.4 | 11.67 | 3.5 | 8 | 0.17 | 2.9 | 3.47 | 2.9 | 11.67 | 7.48 | 960 |
| 0.4 | 12.75 | 4.05 | 8 | 0.7 | 2.35 | 3.47 | 2.37 | 12.75 | 7.58 | 1240 |
| 0.3 | 8.6 | 3.4 | 5.2 | 0 | 3.4 | 3.42 | 3.42 | 8.6 | 6.73 | 661.6 |
| 0.4 | 6.18 | 4.1 | 2.08 | 0 | 2.7 | 3.58 | 2.7 | 6.18 | 4.86 | 480 |
| 0.4 | 11.59 | 3.45 | 8 | 0.14 | 2.95 | 3.52 | 2.98 | 11.59 | 7.48 | 885 |
| 0.4 | 13.35 | 4.35 | 8 | 1 | 2.05 | 3.55 | 2.15 | 13.35 | 7.631086142 | 1344 |
| 0.3 | 8.65 | 3.4 | 5.25 | 0 | 3.4 | 3.49 | 3.44 | 8.65 | 6.75 | 610.7 |
| 0.4 | 11.65 | 3.45 | 8 | 0.2 | 2.95 | 3.5 | 2.9 | 11.65 | 7.52 | 1017.9 |
| 0.4 | 13.41 | 4.35 | 8 | 1.06 | 2.05 | 3.55 | 2.09 | 13.41 | 7.66 | 1321 |
| 0.4 | 11.58 | 3.45 | 8 | 0.13 | 2.95 | 3.48 | 2.95 | 11.58 | 7.48 | 1240 |
| 0.3 | 8.65 | 3.4 | 5.25 | 0 | 3.4 | 3.48 | 3.43 | 8.65 | 6.75 | 555.4 |
| 0.4 | 10.64 | 3.4 | 7.24 | 0 | 3.4 | 3.44 | 3.4 | 10.64 | 7.26 | 1395 |
| 0.4 | 10.75 | 3.4 | 7.35 | 0 | 3.4 | 3.55 | 3.4 | 10.75 | 7.29 | 1017.9 |
| 0.3 | 8.65 | 3.4 | 5.25 | 0 | 3.4 | 3.45 | 3.4 | 8.65 | 6.75 | 610.7 |
| 0.4 | 12.95 | 4.35 | 8 | 0.6 | 2.05 | 3.4 | 2.4 | 12.95 | 7.4 | 1473 |
| 0.3 | 8.65 | 3.4 | 5.25 | 0 | 3.4 | 3.46 | 3.41 | 8.65 | 6.75 | 559.8 |
| 0.3 | 8.65 | 3.4 | 5.25 | 0 | 3.4 | 3.47 | 3.42 | 8.65 | 6.75 | 610.7 |
| 0.4 | 11.72 | 3.45 | 8 | 0.27 | 2.95 | 3.65 | 2.98 | 11.72 | 7.57 | 1248 |
| 0.4 | 11.7 | 3.5 | 8 | 0.2 | 2.9 | 3.51 | 2.91 | 11.7 | 7.5 | 960 |
| 0.4 | 6.1 | 4.1 | 2 | 0 | 2.7 | 3.52 | 2.72 | 6.1 | 4.795081967 | 610.7 |
| 0.3 | 8.7 | 3.4 | 5.3 | 0 | 3.4 | 3.51 | 3.41 | 8.7 | 6.76 | 559.8 |
| 0.3 | 8.62 | 3.4 | 5.22 | 0 | 3.4 | 3.45 | 3.43 | 8.62 | 6.738979118 | 559.8 |
| 0.4 | 13.44 | 4.35 | 8 | 1.09 | 2.05 | 3.57 | 2.08 | 13.44 | 7.68 | 1395 |
| 0.4 | 13.55 | 4.45 | 8 | 1.1 | 1.95 | 3.46 | 1.96 | 13.55 | 7.65 | 1248 |
| 0.3 | 8.6 | 3.4 | 5.2 | 0 | 3.4 | 3.42 | 3.42 | 8.6 | 6.73 | 559.8 |
| 0.3 | 8.6 | 3.4 | 5.2 | 0 | 3.4 | 3.44 | 3.44 | 8.6 | 6.73 | 661.6 |
| 0.3 | 8.63 | 3.4 | 5.23 | 0 | 3.4 | 3.45 | 3.42 | 8.63 | 6.74 | 559.8 |
| 0.4 | 13.4 | 4.35 | 8 | 1.05 | 2.05 | 3.54 | 2.09 | 13.4 | 7.66 | 1224.8 |
| 0.4 | 6.15 | 4.1 | 2.05 | 0 | 2.7 | 3.58 | 2.73 | 6.15 | 4.833333333 | 661.6 |
| 0.4 | 13.63 | 4.45 | 8 | 1.18 | 1.95 | 3.58 | 2 | 13.63 | 7.690755686 | 1032.4 |
| 0.3 | 8.6 | 3.4 | 5.2 | 0 | 3.4 | 3.43 | 3.43 | 8.6 | 6.73 | 661.6 |
| 0.3 | 8.65 | 3.4 | 5.25 | 0 | 3.4 | 3.49 | 3.44 | 8.65 | 6.75 | 661.6 |
| 0.4 | 13.27 | 4.25 | 8 | 1.02 | 2.15 | 3.58 | 2.16 | 13.27 | 7.680859081 | 1248 |
| 0.4 | 12.45 | 4.35 | 8 | 0.1 | 2.05 | 3.4 | 2.9 | 12.45 | 7.1 | 1473 |
| 0.4 | 10.7 | 3.4 | 7.3 | 0 | 3.4 | 3.61 | 3.51 | 10.7 | 7.28 | 1115.2 |
| 0.4 | 11.6 | 3.45 | 8 | 0.15 | 2.95 | 3.64 | 3.09 | 11.6 | 7.49 | 1344 |
| 0.4 | 11.05 | 3.85 | 7.2 | 0 | 2.95 | 3.57 | 3.57 | 11.05 | 7.06 | 1440 |
| 0.4 | 13.35 | 4.35 | 8 | 1 | 2.05 | 3.48 | 2.08 | 13.35 | 7.631086142 | 1395 |
| 0.4 | 11.35 | 3.85 | 7.5 | 0 | 2.95 | 3.68 | 3.38 | 11.35 | 7.13 | 1425 |
| 0.3 | 8.7 | 3.4 | 5.3 | 0 | 3.4 | 3.52 | 3.42 | 8.7 | 6.76 | 508.9 |
| 0.3 | 8.6 | 3.4 | 5.2 | 0 | 3.4 | 3.45 | 3.45 | 8.6 | 6.73 | 610.7 |
| 0.3 | 8.65 | 3.4 | 5.25 | 0 | 3.4 | 3.47 | 3.42 | 8.65 | 6.75 | 610.7 |
| 0.4 | 13.42 | 4.35 | 8 | 1.07 | 2.05 | 3.56 | 2.09 | 13.42 | 7.67 | 1224.8 |
| 0.3 | 8.65 | 3.4 | 5.25 | 0 | 3.4 | 3.49 | 3.44 | 8.65 | 6.75 | 661.6 |
| 0.4 | 13.55 | 4.45 | 8 | 1.1 | 1.95 | 3.49 | 1.99 | 13.55 | 7.65 | 1128.6 |
| 0.4 | 10.71 | 3.4 | 7.31 | 0 | 3.4 | 3.56 | 3.45 | 10.71 | 7.277777778 | 1032.4 |
| 0.4 | 11.57 | 3.45 | 8 | 0.12 | 2.95 | 3.47 | 2.95 | 11.57 | 7.47 | 1318 |
| 0.3 | 8.7 | 3.4 | 5.3 | 0 | 3.4 | 3.5 | 3.4 | 8.7 | 6.76 | 610.7 |
| 0.4 | 13.35 | 4.35 | 8 | 1 | 2.05 | 3.45 | 2.05 | 13.35 | 7.631086142 | 1248 |
| 0.4 | 13.3 | 4.35 | 8 | 0.95 | 2.05 | 3.44 | 2.09 | 13.3 | 7.603383459 | 1152 |
| 0.3 | 8.65 | 3.4 | 5.25 | 0 | 3.4 | 3.46 | 3.41 | 8.65 | 6.75 | 559.8 |
| 0.4 | 13.15 | 4.35 | 8 | 0.8 | 2.05 | 3.46 | 2.26 | 13.15 | 7.52 | 1395 |
| 0.4 | 13.41 | 4.35 | 8 | 1.06 | 2.05 | 3.56 | 2.1 | 13.41 | 7.66 | 1321 |
| 0.4 | 12.75 | 4.05 | 8 | 0.7 | 2.35 | 3.47 | 2.37 | 12.75 | 7.58 | 1318 |
| 0.4 | 13.3 | 4.35 | 8 | 0.95 | 2.05 | 3.48 | 2.13 | 13.3 | 7.6 | 1392 |
| 0.4 | 11.65 | 3.45 | 8 | 0.2 | 2.95 | 3.53 | 2.93 | 11.65 | 7.52 | 1068.8 |
| 0.3 | 8.64 | 3.4 | 5.24 | 0 | 3.4 | 3.45 | 3.41 | 8.64 | 6.75 | 610.7 |
| 0.3 | 8.67 | 3.4 | 5.27 | 0 | 3.4 | 3.51 | 3.44 | 8.67 | 6.754901961 | 661.6 |
| 0.4 | 12.35 | 4.75 | 7.6 | 0 | 2.05 | 3.44 | 3.04 | 12.35 | 6.807692308 | 1473 |
| 0.4 | 13.15 | 4.25 | 8 | 0.9 | 2.15 | 3.56 | 2.26 | 13.15 | 7.61 | 1395 |
| 0.4 | 13.55 | 4.35 | 8 | 1.2 | 2.05 | 3.62 | 2.02 | 13.55 | 7.74 | 1119.7 |
| 0.3 | 8.65 | 3.4 | 5.25 | 0 | 3.4 | 3.48 | 3.43 | 8.65 | 6.75 | 559.8 |
| 0.3 | 8.7 | 3.4 | 5.3 | 0 | 3.4 | 3.52 | 3.42 | 8.7 | 6.764367816 | 508.9 |
| 0.3 | 8.65 | 3.4 | 5.25 | 0 | 3.4 | 3.49 | 3.44 | 8.65 | 6.75 | 559.8 |
| 0.4 | 10.8 | 3.4 | 7.4 | 0 | 3.4 | 3.62 | 3.42 | 10.8 | 7.296296296 | 1017.9 |
| 0.4 | 10.7 | 3.4 | 7.3 | 0 | 3.4 | 3.5 | 3.4 | 10.7 | 7.28 | 1056 |
| 0.3 | 8.6 | 3.4 | 5.2 | 0 | 3.4 | 3.43 | 3.43 | 8.6 | 6.73 | 559.8 |
| 0.3 | 8.65 | 3.4 | 5.25 | 0 | 3.4 | 3.46 | 3.41 | 8.65 | 6.75 | 610.7 |
| 0.4 | 11.15 | 3.85 | 7.3 | 0 | 2.95 | 3.68 | 3.58 | 11.15 | 7.082959641 | 1440 |
| 0.3 | 8.6 | 3.4 | 5.2 | 0 | 3.4 | 3.43 | 3.43 | 8.6 | 6.73 | 508.9 |
| 0.4 | 13.43 | 4.35 | 8 | 1.08 | 2.05 | 3.53 | 2.05 | 13.43 | 7.67 | 1248 |
| 0.4 | 13.31 | 4.35 | 8 | 0.96 | 2.05 | 3.43 | 2.07 | 13.31 | 7.608940646 | 1119.7 |
| 0.4 | 10.7 | 3.4 | 7.3 | 0 | 3.4 | 3.5 | 3.4 | 10.7 | 7.275700935 | 900 |
| 0.4 | 13.38 | 4.35 | 8 | 1.03 | 2.05 | 3.53 | 2.1 | 13.38 | 7.65 | 1395 |
| 0.4 | 13.42 | 4.35 | 8 | 1.07 | 2.05 | 3.52 | 2.05 | 13.42 | 7.67 | 1425 |
| 0.3 | 8.6 | 3.4 | 5.2 | 0 | 3.4 | 3.43 | 3.43 | 8.6 | 6.73 | 661.6 |
| 0.3 | 8.69 | 3.4 | 5.29 | 0 | 3.4 | 3.54 | 3.45 | 8.69 | 6.76 | 661.6 |
| 0.4 | 11.09 | 3.85 | 7.24 | 0 | 2.95 | 3.62 | 3.58 | 11.09 | 7.069882777 | 1344 |
| 0.3 | 8.63 | 3.4 | 5.23 | 0 | 3.4 | 3.44 | 3.41 | 8.63 | 6.74 | 610.7 |
| 0.3 | 8.62 | 3.4 | 5.22 | 0 | 3.4 | 3.44 | 3.42 | 8.62 | 6.74 | 559.8 |
| 0.3 | 8.64 | 3.4 | 5.24 | 0 | 3.4 | 3.48 | 3.44 | 8.64 | 6.75 | 559.8 |
| 0.3 | 8.7 | 3.4 | 5.3 | 0 | 3.4 | 3.52 | 3.42 | 8.7 | 6.76 | 559.8 |
| 0.4 | 6.06 | 4.1 | 1.96 | 0 | 2.7 | 3.49 | 2.73 | 6.06 | 4.76 | 508.9 |
| 0.3 | 8.65 | 3.4 | 5.25 | 0 | 3.4 | 3.48 | 3.43 | 8.65 | 6.75 | 610.7 |
| 0.4 | 13.45 | 4.35 | 8 | 1.1 | 2.05 | 3.6 | 2.1 | 13.45 | 7.69 | 1297.8 |
| 0.3 | 8.62 | 3.4 | 5.22 | 0 | 3.4 | 3.44 | 3.42 | 8.62 | 6.74 | 661.6 |
| 0.3 | 8.6 | 3.4 | 5.2 | 0 | 3.4 | 3.43 | 3.43 | 8.6 | 6.73 | 661.6 |
| 0.3 | 8.65 | 3.4 | 5.25 | 0 | 3.4 | 3.48 | 3.43 | 8.65 | 6.75 | 610.7 |
| 0.4 | 11.05 | 3.85 | 7.2 | 0 | 2.95 | 3.56 | 3.56 | 11.05 | 7.06 | 1440 |
| 0.3 | 8.63 | 3.4 | 5.23 | 0 | 3.4 | 3.45 | 3.42 | 8.63 | 6.74 | 559.8 |
| 0.3 | 8.67 | 3.4 | 5.27 | 0 | 3.4 | 3.49 | 3.42 | 8.67 | 6.75 | 610.7 |
| 0.3 | 8.65 | 3.4 | 5.25 | 0 | 3.4 | 3.49 | 3.44 | 8.65 | 6.748554913 | 559.8 |
| 0.4 | 13.38 | 4.35 | 8 | 1.03 | 2.05 | 3.51 | 2.08 | 13.38 | 7.647608371 | 1224.8 |
| 0.3 | 8.6 | 3.4 | 5.2 | 0 | 3.4 | 3.41 | 3.41 | 8.6 | 6.73 | 610.7 |
| 0.3 | 8.7 | 3.4 | 5.3 | 0 | 3.4 | 3.5 | 3.4 | 8.7 | 6.76 | 661.6 |
| 0.4 | 10.64 | 3.4 | 7.24 | 0 | 3.4 | 3.44 | 3.4 | 10.64 | 7.26 | 967 |
| 0.4 | 13.3 | 4.35 | 8 | 0.95 | 2.05 | 3.45 | 2.1 | 13.3 | 7.603383459 | 1224.8 |
| 0.4 | 10.62 | 3.4 | 7.22 | 0 | 3.4 | 3.45 | 3.43 | 10.62 | 7.26 | 1240 |
| 0.3 | 8.65 | 3.4 | 5.25 | 0 | 3.4 | 3.49 | 3.44 | 8.65 | 6.75 | 559.8 |
| 0.3 | 8.65 | 3.4 | 5.25 | 0 | 3.4 | 3.47 | 3.42 | 8.65 | 6.75 | 559.8 |
| 0.4 | 6.1 | 4.1 | 2 | 0 | 2.7 | 3.52 | 2.72 | 6.1 | 4.8 | 610.7 |
| 0.4 | 11.75 | 3.5 | 8 | 0.25 | 2.9 | 3.57 | 2.92 | 11.75 | 7.53 | 1056 |
| 0.3 | 8.67 | 3.4 | 5.27 | 0 | 3.4 | 3.5 | 3.43 | 8.67 | 6.754901961 | 610.7 |
| 0.4 | 11.6 | 3.45 | 8 | 0.15 | 2.95 | 3.51 | 2.96 | 11.6 | 7.49 | 1017.9 |
| 0.3 | 8.6 | 3.4 | 5.2 | 0 | 3.4 | 3.42 | 3.42 | 8.6 | 6.73 | 661.6 |
| 0.4 | 13.49 | 4.45 | 8 | 1.04 | 1.95 | 3.43 | 1.99 | 13.49 | 7.614899926 | 1128.6 |
| 0.4 | 12.45 | 4.35 | 8 | 0.1 | 2.05 | 3.48 | 2.98 | 12.45 | 7.098393574 | 1392 |
| 0.4 | 10.63 | 3.4 | 7.23 | 0 | 3.4 | 3.43 | 3.4 | 10.63 | 7.26 | 960 |
| 0.4 | 11.51 | 3.45 | 8 | 0.06 | 2.95 | 3.41 | 2.95 | 11.51 | 7.43 | 1240 |
| 0.3 | 8.65 | 3.4 | 5.25 | 0 | 3.4 | 3.45 | 3.4 | 8.65 | 6.75 | 610.7 |
| 0.3 | 8.6 | 3.4 | 5.2 | 0 | 3.4 | 3.4 | 3.4 | 8.6 | 6.73255814 | 559.8 |
| 0.3 | 8.63 | 3.4 | 5.23 | 0 | 3.4 | 3.45 | 3.42 | 8.63 | 6.74 | 559.8 |
| 0.3 | 8.7 | 3.4 | 5.3 | 0 | 3.4 | 3.5 | 3.4 | 8.7 | 6.76 | 559.8 |
| 0.4 | 13.39 | 4.35 | 8 | 1.04 | 2.05 | 3.49 | 2.05 | 13.39 | 7.65 | 1344 |
| 0.4 | 12.25 | 4.75 | 7.5 | 0 | 2.05 | 3.6 | 3.3 | 12.25 | 6.79 | 1425 |
| 0.4 | 13.46 | 4.35 | 8 | 1.11 | 2.05 | 3.6 | 2.09 | 13.46 | 7.69 | 1128.6 |
| 0.3 | 8.62 | 3.4 | 5.22 | 0 | 3.4 | 3.45 | 3.43 | 8.62 | 6.74 | 559.8 |
| 0.4 | 13.35 | 4.35 | 8 | 1 | 2.05 | 3.45 | 2.05 | 13.35 | 7.63 | 1119.7 |
| 0.3 | 8.65 | 3.4 | 5.25 | 0 | 3.4 | 3.46 | 3.41 | 8.65 | 6.75 | 661.6 |
| 0.4 | 13.3 | 4.35 | 8 | 0.95 | 2.05 | 3.45 | 2.1 | 13.3 | 7.6 | 1395 |
| 0.3 | 8.64 | 3.4 | 5.24 | 0 | 3.4 | 3.48 | 3.44 | 8.64 | 6.74537037 | 610.7 |
| 0.4 | 11.65 | 3.45 | 8 | 0.2 | 2.95 | 3.61 | 3.01 | 11.65 | 7.52 | 1344 |
| 0.4 | 13.4 | 4.35 | 8 | 1.05 | 2.05 | 3.5 | 4.35 | 13.4 | 7.66 | 1297.8 |
| 0.3 | 8.6 | 3.4 | 5.2 | 0 | 3.4 | 3.42 | 3.42 | 8.6 | 6.73 | 610.7 |
| 0.4 | 13.4 | 4.35 | 8 | 1.05 | 2.05 | 3.55 | 2.1 | 13.4 | 7.66 | 1224.8 |
| 0.3 | 8.62 | 3.4 | 5.22 | 0 | 3.4 | 3.45 | 3.43 | 8.62 | 6.74 | 559.8 |
| 0.3 | 8.63 | 3.4 | 5.23 | 0 | 3.4 | 3.46 | 3.43 | 8.63 | 6.74 | 559.8 |
| 0.3 | 8.7 | 3.4 | 5.3 | 0 | 3.4 | 3.51 | 3.41 | 8.7 | 6.76 | 559.8 |
| 0.3 | 8.65 | 3.4 | 5.25 | 0 | 3.4 | 3.48 | 3.43 | 8.65 | 6.75 | 559.8 |
| 0.4 | 6.1 | 4.1 | 2 | 0 | 2.7 | 3.54 | 2.74 | 6.1 | 4.8 | 712.5 |
| 0.4 | 10.8 | 3.4 | 7.4 | 0 | 3.4 | 3.58 | 3.38 | 10.8 | 7.3 | 967 |
| 0.4 | 13.39 | 4.35 | 8 | 1.04 | 2.05 | 3.52 | 2.08 | 13.39 | 7.65 | 1321 |
| 0.4 | 13.25 | 4.35 | 8 | 0.9 | 2.05 | 3.38 | 2.08 | 13.25 | 7.58 | 1395 |
| 0.4 | 10.66 | 3.4 | 7.26 | 0 | 3.4 | 3.48 | 3.42 | 10.66 | 7.27 | 1032.4 |
| 0.4 | 11.65 | 3.45 | 8 | 0.2 | 2.95 | 3.52 | 2.92 | 11.65 | 7.521459227 | 967 |
| 0.3 | 8.64 | 3.4 | 5.24 | 0 | 3.4 | 3.49 | 3.45 | 8.64 | 6.75 | 610.7 |
| 0.3 | 8.7 | 3.4 | 5.3 | 0 | 3.4 | 3.52 | 3.42 | 8.7 | 6.76 | 661.6 |
| 0.3 | 8.62 | 3.4 | 5.22 | 0 | 3.4 | 3.44 | 3.42 | 8.62 | 6.74 | 610.7 |
| 0.4 | 11.56 | 3.45 | 8 | 0.11 | 2.95 | 3.46 | 2.95 | 11.56 | 7.463235294 | 1240 |
| 0.4 | 13.49 | 4.45 | 8 | 1.04 | 1.95 | 3.44 | 2 | 13.49 | 7.61 | 1128.6 |
| 0.4 | 10.7 | 3.4 | 7.3 | 0 | 3.4 | 3.49 | 3.39 | 10.7 | 7.28 | 1068.8 |
| 0.3 | 8.64 | 3.4 | 5.24 | 0 | 3.4 | 3.45 | 3.41 | 8.64 | 6.75 | 661.6 |
| 0.4 | 8.94 | 3.55 | 5.39 | 0 | 3.25 | 3.44 | 3.25 | 8.94 | 6.72 | 1083 |
| 0.4 | 10.67 | 3.4 | 7.27 | 0 | 3.4 | 3.49 | 3.42 | 10.67 | 7.27 | 1248 |
| 0.4 | 13.44 | 4.35 | 8 | 1.09 | 2.05 | 3.58 | 2.09 | 13.44 | 7.680431548 | 1224.8 |
| 0.3 | 8.66 | 3.4 | 5.26 | 0 | 3.4 | 3.51 | 3.45 | 8.66 | 6.75 | 610.7 |
| 0.4 | 10.75 | 3.4 | 7.35 | 0 | 3.4 | 3.57 | 3.42 | 10.75 | 7.29 | 1119.7 |
| 0.3 | 8.67 | 3.4 | 5.27 | 0 | 3.4 | 3.51 | 3.44 | 8.67 | 6.75 | 661.6 |
| 0.3 | 8.6 | 3.4 | 5.2 | 0 | 3.4 | 3.4 | 3.4 | 8.6 | 6.73 | 559.8 |
| 0.3 | 8.6 | 3.4 | 5.2 | 0 | 3.4 | 3.42 | 3.42 | 8.6 | 6.73255814 | 610.7 |
| 0.4 | 13.4 | 4.35 | 8 | 1.05 | 2.05 | 3.5 | 2.05 | 13.4 | 7.65858209 | 1152 |
| 0.4 | 13.46 | 4.45 | 8 | 1.01 | 1.95 | 3.4 | 1.99 | 13.46 | 7.6 | 1128.6 |
| 0.4 | 10.66 | 3.4 | 7.26 | 0 | 3.4 | 3.46 | 3.4 | 10.66 | 7.27 | 1152 |
| 0.4 | 11.55 | 3.45 | 8 | 0.1 | 2.95 | 3.6 | 3.1 | 11.55 | 7.46 | 1344 |
| 0.3 | 8.65 | 3.4 | 5.25 | 0 | 3.4 | 3.48 | 3.43 | 8.65 | 6.748554913 | 559.8 |
| 0.4 | 13.43 | 4.35 | 8 | 1.08 | 2.05 | 3.58 | 2.1 | 13.43 | 7.67 | 1224.8 |
| 0.3 | 8.65 | 3.4 | 5.25 | 0 | 3.4 | 3.47 | 3.42 | 8.65 | 6.75 | 559.8 |
| 0.3 | 8.6 | 3.4 | 5.2 | 0 | 3.4 | 3.42 | 3.42 | 8.6 | 6.73 | 610.7 |
| 0.4 | 13.36 | 4.35 | 8 | 1.01 | 2.05 | 3.46 | 2.05 | 13.36 | 7.64 | 1550 |
| 0.3 | 8.64 | 3.4 | 5.24 | 0 | 3.4 | 3.46 | 3.42 | 8.64 | 6.75 | 610.7 |
| 0.4 | 11.95 | 4.75 | 7.2 | 0 | 2.05 | 3.68 | 3.68 | 11.95 | 6.72 | 1344 |
| 0.4 | 9.74 | 3.45 | 6.29 | 0 | 3.35 | 3.44 | 3.35 | 9.74 | 7.02 | 1240 |
| 0.4 | 13.22 | 4.35 | 8 | 0.87 | 2.05 | 3.37 | 2.1 | 13.22 | 7.558623298 | 1473 |
| 0.3 | 8.58 | 3.4 | 5.18 | 0 | 3.4 | 3.38 | 3.4 | 8.58 | 6.73 | 559.8 |
| 0.4 | 13.49 | 4.45 | 8 | 1.04 | 1.95 | 3.43 | 1.99 | 13.49 | 7.614899926 | 1224.8 |
| 0.4 | 10.64 | 3.4 | 7.24 | 0 | 3.4 | 3.44 | 3.4 | 10.64 | 7.26 | 1240 |
| 0.4 | 13.15 | 4.35 | 8 | 0.8 | 2.05 | 3.45 | 2.25 | 13.15 | 7.519011407 | 1392 |
| 0.4 | 12 | 4.75 | 7.25 | 0 | 2.05 | 3.65 | 3.6 | 12 | 6.73 | 1425 |
| 0.4 | 13.38 | 4.35 | 8 | 1.03 | 2.05 | 3.48 | 2.05 | 13.38 | 7.65 | 1318 |
| 0.4 | 11.7 | 3.45 | 8 | 0.25 | 2.95 | 3.64 | 2.99 | 11.7 | 7.55 | 1119.7 |
| 0.4 | 13.06 | 4.2 | 8 | 0.86 | 2.2 | 3.48 | 2.22 | 13.06 | 7.61 | 1344 |
| 0.3 | 8.65 | 3.4 | 5.25 | 0 | 3.4 | 3.47 | 3.42 | 8.65 | 6.75 | 559.8 |
| 0.4 | 13.35 | 4.35 | 8 | 1 | 2.05 | 3.55 | 2.15 | 13.35 | 7.63 | 1344 |
| 0.3 | 8.62 | 3.4 | 5.22 | 0 | 3.4 | 3.42 | 3.4 | 8.62 | 6.74 | 508.9 |
| 0.3 | 8.63 | 3.4 | 5.23 | 0 | 3.4 | 3.46 | 3.43 | 8.63 | 6.74 | 661.6 |
| 0.4 | 11.59 | 3.45 | 8 | 0.14 | 2.95 | 3.52 | 2.98 | 11.59 | 7.482743745 | 885 |
| 0.4 | 10.8 | 3.4 | 7.4 | 0 | 3.4 | 3.61 | 3.41 | 10.8 | 7.296296296 | 1152 |
| 0.4 | 12.25 | 4.75 | 7.5 | 0 | 2.05 | 3.45 | 3.15 | 12.25 | 6.79 | 1297.8 |
| 0.4 | 12.15 | 4.65 | 7.5 | 0 | 2.15 | 3.55 | 3.25 | 12.15 | 6.82 | 1551 |
| 0.3 | 8.6 | 3.4 | 5.2 | 0 | 3.4 | 3.44 | 3.44 | 8.6 | 6.73 | 559.8 |
| 0.4 | 12.4 | 4.35 | 8 | 0.05 | 2.05 | 3.58 | 3.13 | 12.4 | 7.07 | 1344 |
| 0.4 | 13.43 | 4.35 | 8 | 1.08 | 2.05 | 3.53 | 2.05 | 13.43 | 7.67 | 1152 |
| 0.3 | 8.65 | 3.4 | 5.25 | 0 | 3.4 | 3.47 | 3.42 | 8.65 | 6.75 | 559.8 |
| 0.4 | 6.11 | 4.1 | 2.01 | 0 | 2.7 | 3.53 | 2.72 | 6.11 | 4.8 | 528 |
| 0.4 | 13.35 | 4.35 | 8 | 1 | 2.05 | 3.5 | 2.1 | 13.35 | 7.63 | 1395 |
| 0.4 | 10.75 | 3.4 | 7.35 | 0 | 3.4 | 3.57 | 3.42 | 10.75 | 7.29 | 1068.8 |
| 0.3 | 8.7 | 3.4 | 5.3 | 0 | 3.4 | 3.5 | 3.4 | 8.7 | 6.76 | 559.8 |
| 0.4 | 13.33 | 4.35 | 8 | 0.98 | 2.05 | 3.53 | 2.15 | 13.33 | 7.620030008 | 1395 |
| 0.3 | 8.7 | 3.4 | 5.3 | 0 | 3.4 | 3.5 | 3.4 | 8.7 | 6.76 | 610.7 |
| 0.4 | 6.18 | 4.1 | 2.08 | 0 | 2.7 | 3.63 | 2.75 | 6.18 | 4.855987055 | 432 |
| 0.3 | 8.65 | 3.4 | 5.25 | 0 | 3.4 | 3.48 | 3.43 | 8.65 | 6.75 | 508.9 |
| 0.3 | 8.58 | 3.4 | 5.18 | 0 | 3.4 | 3.38 | 3.4 | 8.58 | 6.73 | 559.8 |
| 0.3 | 8.7 | 3.4 | 5.3 | 0 | 3.4 | 3.52 | 3.42 | 8.7 | 6.76 | 610.7 |
| 0.4 | 12.15 | 4.65 | 7.5 | 0 | 2.15 | 3.59 | 3.29 | 12.15 | 6.82 | 1551 |
| 0.4 | 13.36 | 4.35 | 8 | 1.01 | 2.05 | 3.46 | 2.05 | 13.36 | 7.64 | 1318 |
| 0.4 | 11.67 | 3.45 | 8 | 0.22 | 2.95 | 3.57 | 2.95 | 11.67 | 7.534275921 | 1344 |
| 0.3 | 8.65 | 3.4 | 5.25 | 0 | 3.4 | 3.46 | 3.41 | 8.65 | 6.75 | 610.7 |
| 0.4 | 13.35 | 4.35 | 8 | 1 | 2.05 | 3.5 | 2.1 | 13.35 | 7.63 | 1224.8 |
| 0.4 | 13.42 | 4.35 | 8 | 1.07 | 2.05 | 3.52 | 2.05 | 13.42 | 7.6695231 | 1425 |
| 0.4 | 8.7 | 3.45 | 5.25 | 0 | 3.35 | 3.45 | 3.4 | 8.7 | 6.72 | 1240 |
| 0.3 | 8.65 | 3.4 | 5.25 | 0 | 3.4 | 3.46 | 3.41 | 8.65 | 6.75 | 610.7 |
| 0.3 | 8.65 | 3.4 | 5.25 | 0 | 3.4 | 3.46 | 3.41 | 8.65 | 6.75 | 559.8 |
| 0.3 | 8.65 | 3.4 | 5.25 | 0 | 3.4 | 3.48 | 3.43 | 8.65 | 6.75 | 508.9 |
| 0.4 | 13.32 | 4.35 | 8 | 0.97 | 2.05 | 3.42 | 2.05 | 13.32 | 7.614489489 | 1317 |
| 0.3 | 8.68 | 3.4 | 5.28 | 0 | 3.4 | 3.5 | 3.42 | 8.68 | 6.76 | 661.6 |
| 0.3 | 8.66 | 3.4 | 5.26 | 0 | 3.4 | 3.47 | 3.41 | 8.66 | 6.751732102 | 559.8 |
| 0.4 | 11.2 | 3.85 | 7.35 | 0 | 2.95 | 3.64 | 3.49 | 11.2 | 7.09 | 1425 |
| 0.4 | 13.4 | 4.35 | 8 | 1.05 | 2.05 | 3.51 | 2.06 | 13.4 | 7.65858209 | 1344 |
| 0.3 | 8.7 | 3.4 | 5.3 | 0 | 3.4 | 3.54 | 3.44 | 8.7 | 6.76 | 661.6 |
| 0.4 | 11.4 | 3.85 | 7.55 | 0 | 2.95 | 3.63 | 3.28 | 11.4 | 7.14 | 1440 |
| 0.4 | 13.3 | 4.35 | 8 | 0.95 | 2.05 | 3.41 | 2.06 | 13.3 | 7.603383459 | 1323.2 |
| 0.4 | 10.67 | 3.4 | 7.27 | 0 | 3.4 | 3.48 | 3.41 | 10.67 | 7.27 | 1152 |
| 0.4 | 13.25 | 4.35 | 8 | 0.9 | 2.05 | 3.46 | 2.16 | 13.25 | 7.58 | 1395 |
| 0.3 | 8.65 | 3.4 | 5.25 | 0 | 3.4 | 3.49 | 3.44 | 8.65 | 6.75 | 610.7 |
| 0.4 | 11.15 | 3.85 | 7.3 | 0 | 2.95 | 3.7 | 3.6 | 11.15 | 7.082959641 | 1440 |
| 0.4 | 13.3 | 4.35 | 8 | 0.95 | 2.05 | 3.45 | 2.1 | 13.3 | 7.6 | 1221.5 |
| 0.3 | 8.6 | 3.4 | 5.2 | 0 | 3.4 | 3.4 | 3.4 | 8.6 | 6.73 | 610.7 |
| 0.3 | 8.6 | 3.4 | 5.2 | 0 | 3.4 | 3.44 | 3.44 | 8.6 | 6.73 | 611.6 |
| 0.3 | 8.6 | 3.4 | 5.2 | 0 | 3.4 | 3.43 | 3.43 | 8.6 | 6.73 | 559.8 |
| 0.4 | 11.58 | 3.45 | 8 | 0.13 | 2.95 | 3.48 | 2.95 | 11.58 | 7.48 | 1318 |
| 0.3 | 8.67 | 3.4 | 5.27 | 0 | 3.4 | 3.49 | 3.42 | 8.67 | 6.75 | 559.8 |
| 0.4 | 10.7 | 3.4 | 7.3 | 0 | 3.4 | 3.48 | 3.38 | 10.7 | 7.28 | 967 |
| 0.3 | 8.6 | 3.4 | 5.2 | 0 | 3.4 | 3.42 | 3.42 | 8.6 | 6.73 | 559.8 |
| 0.4 | 11.75 | 3.45 | 8 | 0.3 | 2.95 | 3.66 | 2.96 | 11.75 | 7.59 | 960 |
| 0.3 | 8.6 | 3.4 | 5.2 | 0 | 3.4 | 3.38 | 3.38 | 8.6 | 6.73 | 610.7 |
