# Supplementary material for: Pareto-optimized stacked ensemble machine learning framework for predicting bearing capacity of driven piles from static load test data
Source: Sci Rep. 2026 Apr 2;16:11360. doi: 10.1038/s41598-026-43660-z (PMC13049102; doi:10.1038/s41598-026-43660-z)
Supplement: Supplementary file 2 — Supplementary Material 2 [file 41598_2026_43660_MOESM2_ESM.docx]

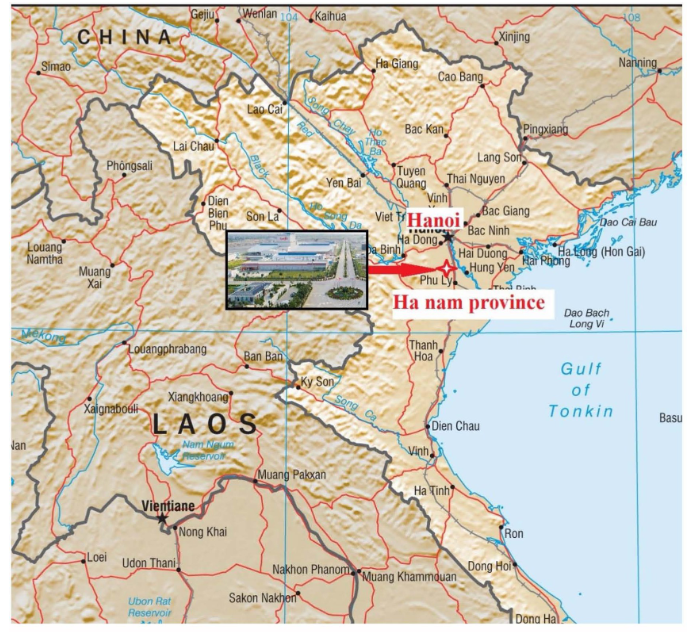


**Supplementary Fig. S1.** Approximate location of the test sites in Ha Nam province, Vietnam (obtained from the key reference Pham et al. [42]).
